# Cell-Penetrating Peptides as Valuable Tools for Nose-to-Brain Delivery of Biological Drugs

**DOI:** 10.3390/cells12121643

**Published:** 2023-06-16

**Authors:** Lisa Benedetta De Martini, Claudia Sulmona, Liliana Brambilla, Daniela Rossi

**Affiliations:** Laboratory for Research on Neurodegenerative Disorders, Istituti Clinici Scientifici Maugeri-IRCCS, 27100 Pavia, Italy; lisa.dema.ldm@gmail.com (L.B.D.M.); claudia.sulmona@icsmaugeri.it (C.S.); liliana.brambilla@icsmaugeri.it (L.B.)

**Keywords:** cell-penetrating peptides, nose-to-brain administration, rehabilitation, therapy

## Abstract

Due to their high specificity toward the target and their low toxicity, biological drugs have been successfully employed in a wide range of therapeutic areas. It is yet to be mentioned that biologics exhibit unfavorable pharmacokinetic properties, are susceptible to degradation by endogenous enzymes, and cannot penetrate biological barriers such as the blood–brain barrier (i.e., the major impediment to reaching the central nervous system (CNS)). Attempts to overcome these issues have been made by exploiting the intracerebroventricular and intrathecal routes of administration. The invasiveness and impracticality of these procedures has, however, prompted the development of novel drug delivery strategies including the intranasal route of administration. This represents a non-invasive way to achieve the CNS, reducing systemic exposure. Nonetheless, biotherapeutics strive to penetrate the nasal epithelium, raising the possibility that direct delivery to the nervous system may not be straightforward. To maximize the advantages of the intranasal route, new approaches have been proposed including the use of cell-penetrating peptides (CPPs) and CPP-functionalized nanosystems. This review aims at describing the most impactful attempts in using CPPs as carriers for the nose-to-brain delivery of biologics by analyzing their positive and negative aspects.

## 1. Introduction

Biological drugs such as proteins, peptides, and oligonucleotides have been successfully employed in a wide range of therapeutic areas including oncology, inflammatory and autoimmune diseases, hemophilia, and rare genetic diseases [1]. Due to their macromolecular nature and structural complexity, they display a higher specificity toward the target and consequentially a lower toxicity when compared to small molecules, which are more promiscuous and prone to inducing non-target effects [2,3]. As a result, biologics are usually well-tolerated and can be transferred to clinical development more easily than small molecules. On the other hand, they display unfavorable pharmacokinetic properties, a feature that limits their therapeutic values [4]. Being essentially composed of natural amino acids or nucleic acids, biotherapeutics are susceptible to degradation by endogenous enzymes such as serum proteases or nucleases, which can limit both their stability in the systemic circulation as well as their efficacy after systemic administration [5,6]. Additionally, biopharmaceuticals can be recognized by the immune system, potentially leading to undesired immune responses characterized by the development of anti-drug antibodies. This phenomenon is commonly defined “immunogenicity”, and it is well-known to alter their pharmacokinetics, to determine a loss of efficacy through neutralization, and to increase adverse events [1,7]. Moreover, due to their high molecular weight and hydrophilic nature, biological drugs display low membrane permeability, a feature that can prevent their penetration across biological barriers including skin as well as mucosal and cell membranes. As a result, biologicals are extremely complex to formulate and deliver, and thereby require parenteral administration in most cases [3]. Even more challenging is the development of biotherapeutics targeting disorders of the central nervous system (CNS), which imply their passage through the blood–brain barrier (BBB) in order to reach the affected nervous tissue [4,8].

The BBB represents the main impediment to deliver systemically administered therapeutics to the CNS. Being mainly composed by endothelial cells of brain capillaries, which are interconnected through tight junctions (TJ), the BBB exhibits a low rate of pinocytosis as well as restricted paracellular transport. Moreover, degrading enzymes and efflux systems such as P-glycoprotein and multidrug resistance proteins (MRPs) further restrict the brain-entry of exogenous substances [4,9,10], making the BBB impermeable to 98% of small molecules and to the totality of macromolecular drugs [11]. Generally, a molecule needs to have a molecular mass under 400–500 Da as well as a great lipophilicity to appreciably cross the BBB via transcellular passive diffusion. Alternatively, it must be recognized by specific transporters or receptors such as glucose transporter 1 and transferrin receptor, which convey into the CNS molecules and are essential for brain function [9,10,11].

For these reasons, the intracerebroventricular (i.c.v.) and intrathecal administration routes are still widely used to deliver biopharmaceutics to the CNS, despite their invasiveness. For instance, Spinraza (nusinersen), an antisense oligonucleotide recently approved by the FDA and EMA for the treatment of 5q spinal muscular atrophy, has been developed for intrathecal use [12,13,14]. Consistently, i.c.v. administration remains the main route through which pharmacological studies of neuropharmaceuticals are performed [15,16,17]. It should, however, be mentioned that these routes of administration present some limitations in terms of treatment allocation (e.g., need for hospitalization), safety, and costs [18]. Thus, other drug delivery strategies have been developed to overcome the obstacle of the BBB and permit the entrance of parentally administered biologicals into the CNS. An option that has been applied consists in the temporary disruption of the BBB following intracarotid arterial infusion of vasoactive agents such as mannitol or DMSO to increase paracellular openings. However, this remains an invasive approach whose safety is uncertain. The temporary disruption of the BBB may in fact allow the entrance into the brain of neurotoxic substances including plasma proteins [5,11]. An additional and less invasive possibility is represented by the generation of ligand- or antibody-fusogenic drugs that can be transported across the BBB by transcytosis upon recognition by a specific receptor (e.g., the transferrin receptor) [5,19]. This approach, however, requires high plasma concentrations of the drug, which may cause adverse effects [20]. Recently, the conjugation of biologics with cell-penetrating peptides (CPPs) was investigated as a promising approach to enhance the passage of biologically active molecules through the BBB after systemic administration. CPPs are short and positively charged peptides (less than 30 amino acids) that are capable of penetrating through plasma and tissue membranes. They are used as delivery carriers to release a variety of biologically active molecules (cargoes) into cells [4,21,22]. However, CPPs display low cell and tissue selectivity, and when administered systemically, the non-specific internalization can result in their accumulation in blood vessels and peripheral organs [19,22], possibly leading to adverse effects.

Moreover, all the strategies listed above require intravenous drug administration, and this may represent a problem in terms of patient compliance, especially for chronic treatments. The intranasal (IN) route of administration provides a non-invasive method to achieve direct delivery of biotherapeutics into the CNS, avoiding the BBB through the olfactory and trigeminal nerve pathways. Thus, the IN route can potentially enable the delivery of neurotherapeutics to the CNS while simultaneously reducing systemic exposure and side effects [10,18]. In addition, medical staff may be no longer be required as patients can self-administer the drugs. Although promising, the IN delivery of biologics comes with some disadvantages. The main limitation is represented by the low nasal drug absorption due to mucociliary clearance and poor nasal mucosal epithelium permeation, resulting in a bioavailability that is below 1% [23]. Various approaches have been introduced to improve nose-to-brain (N2B) drug delivery, one of which is the use of CPPs to increase the drug uptake from the nasal epithelium as well as its penetration into the brain parenchyma.

The aim of the present review is therefore to explore the application of CPPs alone or in combination with polymer and/or lipidic nanocarriers to promote N2B delivery of biologicals.

## 2. CPPs: Chemical Helpers to Improve Membrane Permeability of Biologics

Known also as protein translocation domains, membrane translocation sequences, or Trojan horse peptides [24], CPPs are a family of various peptides, typically comprising 5–30 amino acids (AAs). Noteworthy, they can hold different physical–chemical properties and are united by their capacity to penetrate into cell membranes, biological barriers, and tissues [4]. CPPs can be conjugated with a variety of biologically active cargoes and used as carriers to deliver these molecules into cells. These have been shown to deliver various cargoes including polar macromolecules and nanosystems into different kind of cells, even into difficult-to-transfect cell types such as stem and neuronal cells [24,25]. When used to perform transfection, they display a transfection ability comparable or even greater than those of well-known lipofection reagents such as Lipofectamine 2000 [22]. Moreover, unlike other cellular translocation techniques such as microinjection, electroporation, and amphipathic detergent/viral vector employment, they do not cause cytotoxic effects and can enter the cells in a non-invasive manner [26].

This class of peptides was first introduced in the late 1980s when Frankel et al. [27] discovered the ability of the transactivator of transcription (TAT) protein of HIV-1 to cross cell membranes and to translocate into the nucleus. Later, this translocation capacity was attributed by Vivès et al. [28] to a short sequence of 13 AAs, extending from residue 48 to 60, namely, TAT_48–60_, recognized as the key motif for transduction.

A few years later, penetratin, a 16 AA peptide, derived from the third helix of the homeodomain of *Drosophila Antennapedia*, was also found to induce intracellular penetration [29]. Since the discovery of these internalization features, numerous natural and artificial peptides with cell-penetrating abilities have been identified, synthesized, and combined. Efforts have been made to optimize their sequence and overcome their limits.

No unified taxonomy of these peptides currently exist. Thus, CPPs can be categorized in several ways (i.e., according to (i) their physical–chemical properties; (ii) the interaction between the CPP and the cargo; (iii) their preferred uptake mechanism) [24,26].

## 3. CPP Classification Based on Physical–Chemical Properties

Based on their physical–chemical properties, CPPs can be subdivided into three main categories: cationic, amphipathic, and hydrophobic peptides [4]. The cationic class comprises peptides characterized by an overall positive net charge in physiological conditions. This feature primarily originates from the presence in their sequence of side chains of arginines and lysines, which have a pKa value of 10.3 and 12.5, leading to a positive charge of both amino acids at physiological pH [4,30]. Examples of cationic peptides include first generation CPPs such as TAT-derived moieties, penetratin, polyarginines (R5-R12), but also new generation peptides including low molecular weight protamine (LMWP) and crotamine [24,25] (Table 1).

The two basic AAs, Arg and Lys, are responsible for the strong electrostatic interaction between cationic CPPs and the negatively-charged components of the plasma membrane (e.g., proteoglycans). This prompts an accumulation of the peptide at the level of the membrane and initiates the process of internalization [26,30]. However, Arg can influence the cell-penetrating activity of CPPs differently from Lys. The guanidinium group in the side chain of Arg forms bidentate hydrogen bonds with the phosphate groups of membrane phospholipids. This assures a more stable electrostatic interaction with the cell membrane than the ammonium group of Lys, which can only form a single hydrogen bond. Therefore, Arg-based CPPs are thought to interact more closely with the lipid bilayer than the Lys-based ones, resulting in an easier internalization [30,39]. Among the Arg-based CPPs, oligomers with 6 to 9 Arg (R6–R9) display the best performance in the intracellular delivery of cargoes with minimal toxicity [40] (Table 1).

Amphipathic CPPs are characterized by the presence of both hydrophilic and hydrophobic AAs in their primary structure. This class of peptides can be divided into three subclasses: primary, secondary, and proline-rich CPPs. Primary amphipathic peptides such as MPG or Pep-1 typically contain more than 20 AAs and have separate polar and non-polar sequences of AAs. Secondary amphipathic CPPs such as MAP, the chimeric peptide transportan, and the azurin-derived p28 peptide commonly contain less than 20 AAs and are characterized by intermingled hydrophilic and hydrophobic sequences.

The latter generally assume a characteristic secondary conformation that is essential for their uptake into cells. More specifically, they can form either an α-helical structure, with hydrophilic and hydrophobic residues grouped on different sides of the helix, or a β-sheet structure with defined cationic and hydrophobic faces upon exposure to the membrane environment [4,24,30]. Finally, proline-rich amphipathic CPPs are a plethora of peptides that differ in sequence and structure, but all of which can be distinguished by a proline pyrrolidine template [41]. Examples of proline-rich peptides are represented by synthetic fragments of Bac 7 [4].

The last class of CPPs comprises hydrophobic peptides that mainly contain non-polar residues, resulting in a low net charge. The presence of hydrophobic motifs increases the affinity of these CPPs for the hydrophobic domains of the cellular membrane, thus promoting membrane insertion. Examples of hydrophobic CPPs are the C105Y peptide, its PFVYLI C-terminal portion, and the Pep-7 peptide [4] (Table 1).

## 4. CPP Classification Based on the Type of Coupling to the Cargo

**Figure 1:**
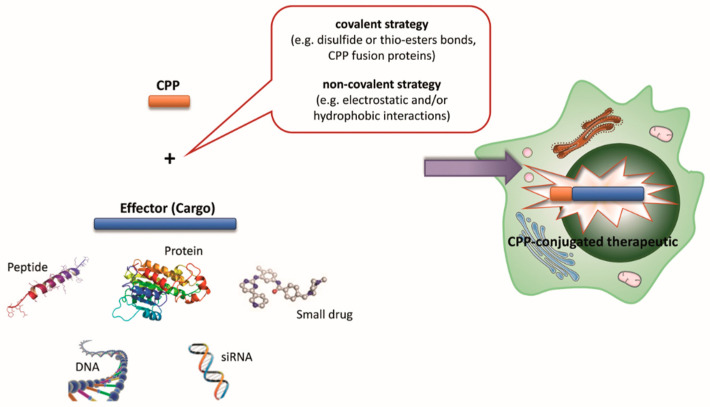
Schematic drawings representing the cell-penetrating peptide (CPP)-based technologies. The hydrophilic nature of effectors/cargoes (blue) such as peptides, proteins, nucleic acids, or small drugs can prevent their cellular uptake and hamper their access to intracellular targets. Conjugating the effector (cargo) to a CPP (orange) by covalent bonds or noncovalent complex formation enables the CPP–effector conjugate (CPP-conjugated therapeutic) to cross the cell membrane and reach intracellular areas that are difficult to access, thereby enhancing the therapeutic effectiveness. Reprinted from Ref. [4]. Copyright 2017, with permission from Elsevier.

According to the type of coupling to the cargo, CPPs can be classified as covalently or non-covalently bound peptides (Figure 1). The former are either fused to a recombinant cargo protein through cloning, thus resulting in a CPP fusion protein, or conjugated by chemical cross-linking to the cargo using bivalent linkers containing a pyridyl or maleinimide moiety on one side and an activated ester on the other. Covalent modifications have been applied to several CPPs such as TAT derivatives, penetratin, and polyarginines (Table 1). Despite not affecting the cell-penetrating activity of the CPPs, covalent coupling has been shown to determine a significant reduction, or even a complete inhibition, of the pharmacological activity of the functional cargo molecules. To overcome this issue, flexible linkers containing degradable bonds (e.g., disulfide or thio-esters bonds) have been introduced between the CPP and the cargo in order to enable the release of the bioactive molecule after delivery to the target [24,26,30] (Figure 1). Covalent coupling results are particularly suitable for charge-neutral oligonucleotides such as peptide nucleic acids (PNAs) and phosphorodiamidate morpholino oligonucleotides (PMOs) [24]. However, it may not be suitable for every situation. For example, CPP–insulin covalent conjugates have been shown to be unstable and to precipitate. Thus, preparing stable CPP–insulin complexes remains quite challenging [42].

The second class of CPPs, according to this classification scheme, is represented by those that are non-covalently bound to their cargo. These include CPPs capable of forming cell-penetrating complexes through electrostatic interactions such as the Pep, MPG, KALA, KLA, and PepFect types (Table 1). When mixed with active molecules that present negative charged regions including plasmid DNA, antisense DNA, and short interfering RNA (siRNA), these peptides are able to form soluble nanoparticles due to their intrinsic positive charges. For example, MPG has been shown to successfully complex and efficiently deliver siRNA into cultured cell lines. One issue that should be, however, considered is that non-covalent CPP–cargo complexes can prematurely release the cargo due to insufficient complex stability [24,26].

## 5. CPP Classification Based on Uptake Mechanism

**Figure 2:**
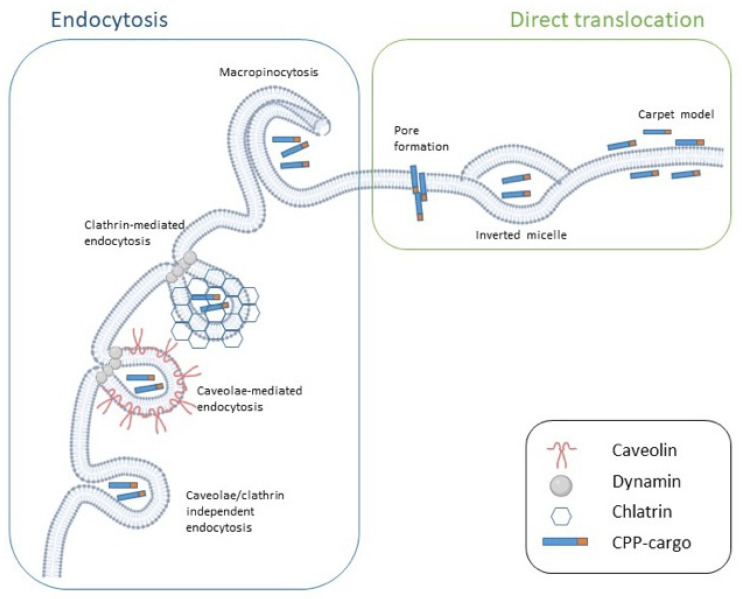
Schematic representation of the proposed mechanisms for CPP internalization. Energy-independent pathways involving the membrane insertion of CPPs through pore formation and membrane destabilization are outlined in green. Energy-dependent models, or endocytic pathways such as macropinocytosis, clathrin-mediated endocytosis, caveolae-mediated endocytosis, and caveolae/clathrin-independent endocytosis are outlined in blue.

CPPs can also be categorized based on their preferred uptake mechanism. Peptide internalization is influenced by a number of parameters including their concentration, target cell, temperature, pH, and cargo type. Even though many variables have been shown to affect CPP uptake, their internalization follows two major routes (i.e., membrane translocation (direct/passive or energy-independent uptake) and endocytosis (active or energy-dependent uptake)) [22,30] (Figure 2).

Membrane translocation, also known as direct penetration, is a one-step, energy-independent mechanism, which occurs at low temperatures and in the presence of endocytotic inhibitors. It requires direct interaction between the peptide and the phospholipid bilayer and is driven by electrostatic forces. Positive-charged regions of CPPs including the guanidine group of the Arg amino acid interact with negative charged components of the plasma membrane such as lipid phosphate groups and heparan sulfates. The peptide hydrophobic face, in the specific case of amphipathic CPPs, interacts with the hydrophobic lipid tails, determining the destabilization of the membrane and resulting in CPP entry [22,26,30]. It is generally accepted that, at physiological pH, direct translocation primarily occurs at a high CPP concentration. Furthermore, it is more probable for primary amphipathic CPPs including transportan analogs and MPG. Penetratin does not meet this rule as it preferentially crosses the membrane in an energy-independent manner at a low concentration, but it switches to endocytosis at a higher concentration [26].

Depending on the type of CPP–membrane interaction, three energy-independent mechanisms have been described: the inverted micelle model, the pore formation model, and the carpet model (Figure 2). Arg-rich peptides tend to form inverted micelles in the membrane environment. More specifically, the formation of electrostatic interactions between the CPP and the membrane leads to changes in the membrane curvature, which, in turn, allows for the formation of inverted micelles. The hydrophilic environment inside the micelle allows for the accumulation of CPP–cargo complexes, which are then released into the cytoplasm [22,26] (Figure 2).

The transient pore formation models are generally proposed as the mechanism preferentially used by amphipathic peptides [26]. For instance, Deshayes et al. [43] reported that Pep-1 can enter the cell via transient pore formation due to its α-helical structure. Direct translocation via pore formation includes two separated models: the barrel-stave and the toroidal models. In the barrel-stave model, upon interaction with the plasma membrane, amphipathic CPPs with an α-helical structure form bundles with a channel at their centers. The hydrophobic part of the peptides is oriented toward the phospholipid bilayer, while the hydrophilic part is oriented toward the center of the channel. On the other hand, in the toroidal model, the interaction between the positive charged face of an amphipathic α-helical CPP and the membrane phosphate groups causes the accumulation of the CPP on the outer leaflet of the membrane, resulting in bending of the lipid bilayer, which forms a pore in the membrane [22,26]. Although this process has been extensively described for α-helical CPPs, MPG, an amphipathic peptide characterized by a β-sheet folding upon interaction with the cargo and phospholipids, enters the cells through the formation of transient pore-like structures [31] (Table 1).

Finally, according to the carpet model, the positively charged portions of CPPs interact with the negatively charged phospholipid headgroups of the membrane, thus determining the self-association of the CPPs in a carpet-like manner, where the peptides lie parallel to the cell membrane instead of inserting perpendicularly into it (Figure 2). This peptide disposition results in a transient increment in membrane fluidity, which allows the CPP translocation. In contrast with the pore formation model, the secondary structure of the peptide does not affect its internalization, which only depends on its orientation. Although electrostatic interactions are essential for the formation of the carpet-like structure, hydrophobic interactions also have an important role in this model to ensure sufficient orientation of the peptide. Examples of CPPs that enter the cell following the carpet model include transportan-10 (TP10) and the amphipathic antimicrobial peptide dermaseptin [4,22,26] (Table 1).

Although membrane translocation was first proposed as the main mechanism for CPP internalization, it is now generally acknowledged that low concentrations as well as conjugation with a cargo (especially large cargoes) favor CPP uptake via endocytosis [4,26]. Endocytosis is an energy-dependent process normally used to internalize macromolecules. It occurs through membrane-bound vesicles derived from the invagination and pinching-off of the plasma membrane [22]. Endocytosis is a multistep process composed of various mechanisms and is generally divided into two major categories (i.e., phagocytosis (the uptake of large particles) and pinocytosis (the uptake of fluids and solutes)). While phagocytosis is normally restricted to specialized cells (e.g., macrophages, monocytes and neutrophils), pinocytosis concerns all mammalian cells.

At least four distinct mechanisms have been described for pinocytosis: macropinocytosis, clathrin-mediated endocytosis (CME), caveolae-mediated endocytosis (CvME), and clathrin- and caveolae-independent endocytosis [44]. CPPs can enter the cell using one or more endocytic pathways depending on the conditions [22] (Figure 2).

Macropinocytosis is a fast, lipid-raft-based mechanism of endocytosis in which no receptor is involved. Various signaling cascades involving the Rho-family GTPases are essential to trigger the actin-driven formation of membrane protrusions that, collapsing onto and fusing with the plasma membrane, give rise to the large endocytic vesicles that sample large volumes of extracellular milieu [26,44] (Figure 2). Macropinocytosis has been proposed as the active uptake mechanism used by several Arg-rich CPPs such as TAT [45] and poly-arginine peptides [46]. To date, it has not been completely elucidated how CPPs can cause such a large-scale remodeling of the plasma membrane [30]. Heparan sulfate proteoglycans and neuropilin 1 have been proposed to play important roles in inducing the macropinocytosis of Arg-rich peptides [47,48].

CME is a receptor-mediated process that depends on clathrin and requires dynamin. In this process, the strong binding of a ligand to a specific cell surface receptor determines the assembly of clathrins in a polyhedral lattice on the cytosolic face of the plasma membrane. This is followed by the invagination of a clathrin-coated membrane surface toward the cytoplasm to form the so-called “coated pits”, which undergo progressive invagination and pinch-off to form clathrin-coated vesicles that carry receptor–ligand complexes into the cell [26,44] (Figure 2). Dynamin, a GTPase protein, usually self-assembles at the neck of the budding vesicle immediately before vesicle fission.

The released clathrin-coated vesicles are soon delivered to early endosomes, which mature to late endosomes and deliver their cargo to lysosomes, where, if not previously released, the cargo is degraded [26]. Thus far, CME has been reported as a pathway used by the unconjugated TAT peptide [32], polyarginines [33], and anionic CPPs such as NickFect1 [34] (Table 1).

Unlike CME, CvME does not necessarily result in the delivery of macromolecules to lysosomes because caveosomes, the structures responsible for intracellular transport in this process, usually deliver their cargos to the Golgi apparatus and the endoplasmic reticulum [22]. Caveolae, from which caveosomes are generated, are flask-shaped invaginations of the plasma membrane, are highly hydrophobic and rich in cholesterol and sfingolipids, in which many signaling molecules and transporters are located [44]. CvME has been described for many CPPs including proline-rich CPPs [41], non-cationic azurin-derived peptides [35], TAT-fusion proteins [36], and transportan–protein complexes [37]. For both transportan and TAT peptides, other uptake mechanisms have also been reported. However, when conjugated with proteins, their preferred internalization pathway seems to be CvME. This could be the result of the increase in size upon conjugation with the cargo [26].

The mechanisms that govern clathrin- and caveolae-independent endocytosis have been reported only in a few cases thus far and remain poorly understood. Azurin and its derived peptides, which were previously mentioned as using CvME to penetrate into cells, were shown to also exploit a caveolae-independent pathway [35]. Moreover, transportan and TP10 were proven to enter the cells via caveosomes, even though another uptake mechanism was shown to be possible [26]. The most recent report of a CPP using this uptake mechanism comes from Ye and collaborators, who found that LMWP/siRNA complexes enter the cell in a clathrin-, caveolae-, and dynamin-independent manner [38] (Table 1).

## 6. Overcoming the Current Limitations of CPPs

The ability of CPPs to deliver poorly permeable drugs even into difficult-to-transfect substrates such as stem and neuronal cells as well as various types of tissue has made CPPs a subject of interest for the scientific community [24,25]. However, despite the promising results achieved by CPPs in in vitro and in vivo studies and the recent increase in the numbers of clinical trials, there are still no CPP-based drugs approved by the principal regulatory agencies [30,49].

The main concerns of CPP-based drugs are represented by their physiological instability, low selectivity, and limited efficacy, principally due to endosomal entrapment. Because of their short peptidic structure, CPPs are particularly susceptible to proteolytic degradation by means of secreted, membrane bound, intracellular, and serum proteases. Moreover, CPPs are rapidly removed from the circulation through renal clearance and via the reticuloendothelial system. The direct consequence of these events is their rapid metabolism, which results in a short half-life upon systemic administration [22,30].

The poor stability of CPPs in vivo depends on elements that influence their proteolytic degradation. These factors include the sequence, conformation, presence of unnatural AAs, D-isomers, and individual chemical bridges, and the routes of administration as well as the type of cargo [6].

Several strategies have been suggested to solve these pharmacokinetic problems including chemical alterations of CPPs. Terminal modifications have been traditionally used to improve peptide endurance because they hinder the enzymatic recognition by exopeptidases. More specifically, N-acetylation and C-terminal amidation have been proposed to enhance CPP stability [30]. Another strategy consists of the replacement of L-AAs by either their D-variants or other unnatural AAs such as ornithine. The stereochemical orientation of the AAs is essential for substrate recognition by proteases. Thus, incorporating D-AAs in the primary structure of CPPs may significantly prolong their half-life in biological fluids. For instance, Purkayastha and colleagues [50] showed how the short half-life of octarginine in human plasma (0.5 min) could be improved by changing the CPP stereochemistry, with the D-form of octarginine having an extended half-life of more than 7 days. This increase in the bioavailability of D-form CPPs was also reported for protein–CPP conjugates. For example, the complex formed by insulin conjugated with D-penetratin, a form of penetratin constituted only by D-AAs, was shown to display a prolonged half-life in rat intestinal fluids when compared to the insulin–L-penetratin complex [51]. However, AA stereochemical alterations can compromise the CPP permeation activity, especially for those CPPs that are characterized by a defined secondary structure. For instance, a single D-AA substitution was reported to dramatically decrease the cellular uptake of the amphipathic azurin-derived p28 CPP in both the cancer and control cell lines [52].

Changing the peptide backbone itself, for example, with a peptoid backbone, is another method to improve CPP stability. Peptoids, or poly-N substituted glycines, are peptidomimetics that evade proteolytic degradation via repositioning the side-chain from the α-carbon to the amide nitrogen. Cell-penetrating peptoids are easier to synthetize and, in some cases, they can even provide a better cellular uptake performance than their native peptides [6,30].

Because proteases can only have limited access to constrained peptides, CPP stability can also be improved by adding conformational constrictions. The more rigid the structure of the peptide, the less prone it is to proteolytic degradation [22]. Any intra- or intermolecular interaction that helps the peptide to adopt a stable folded conformation will enhance the peptide global stability and prevent its enzymatic degradation. In order to reduce CPP conformational flexibility, several strategies based on the introduction of an intramolecular bond have been applied including cyclization and the generation of stapled peptides [6].

Backbone cyclization determines a higher resistance against exopeptidases and proteases by removing the enzymatically vulnerable N- and C-terminals of the peptide. Examples of cyclic peptides are cFΦR4 and sC18, which showed greater proteolytic stability in human serum than their linear correspondents [30]. Not only were cyclic CPPs reported to exhibit higher stability when compared to their linear counterparts, they also seemed to display a more efficient internalization capacity and a greater ability to escape from endosomes [53].

On the other hand, stapled peptides were stabilized by a covalent linkage of two amino acid residues, resulting in the formation of a peptide macrocycle. Thus, a hydrocarbon bridge is created, which increases the α-helicity, stability, target binding affinity, and cell permeability of the peptide. This hydrocarbon bridge partially shields and stabilizes the hydrophilic backbone of the peptide, promotes the interaction between the peptide and the hydrophobic component of the cell membrane, and facilitates cell penetration [22].

Despite the innate ability of some cationic CPPs to escape from endosomes, endosomal entrapment remains one of the limiting factors to achieve efficient cellular uptake of CPP–cargo complexes. A number of different strategies have been developed to ensure cytosol accessibility to those CPPs that are trapped into lysosomes. One approach to promote endosomal escape of CPP-conjugates is the incubation with lysosomotropic reagents such as chloroquine, trifluoromethyl-quinoline, and calcium phosphate [6,24]. The weak base chloroquine can enter the cells upon protonation and accumulate into endosomes. At a low concentration, it inhibits endosome acidification while at a high concentration, it causes endosomal swelling and rupture, thus determining the release of the endosomal cargo [26]. However, lysosomotropic compounds are highly cytotoxic and can therefore only be used in in vitro experiments [22]. Another possibility to overcome this issue is to incorporate a peptide endowed with endosomolytic activity into the structure of a CPP. This can be easily achieved by conjugating the CPP to a pH sensitive fusogenic peptide such as HA2. HA2 is a peptide derived from the hemagglutinin protein of the influenza virus, which undergoes a conformational change upon acid pH exposure, destabilizing the endosome membrane [30]. For instance, a significantly enhanced endosomal escape activity has been reported for HA2-fused TAT compared to TAT alone [45]. In addition to HA2, a synthetic penetration-accelerating sequence (PAS), having the reverse sequence of part of the cathepsin D sequence, has been reported to enhance the efficiency of the cellular uptake of Arg-rich peptides [54]. Besides fusogenic peptides, CPPs can also be conjugated with neutral helper lipids such as dioleoylphosphatidylethanolamine (DOPE), which is able to enhance the release and therapeutic activity of cargos. In addition, DOPE was proven to be capable of improving the transfection efficacy of TAT–pDNA complexes [30]. In the case of specific CPPs such as TP10, N-terminal stearylation was reported to increase internalization and promote endosomal escape. More specifically, stearyl-TP10 was shown to have an enhanced capacity to deliver negatively charged oligonucleotides, similar to the transfection agent Lipofectamine 2000, but exhibiting lower cytotoxicity [55].

A way to enhance the efficacy of CPP internalization bypassing endosomal entrapment is to promote direct translocation by increasing the interactions of CPPs with the inner hydrophobic portion of the membrane. Incubation with amphipathic counteranions (e.g., pyrenebutyrate) [56], conjugation with hydrophobic groups (e.g., hexanoyl) [57], and acylation with fatty acids of different lengths and saturation grades [58] are all strategies that have been proposed to enhance the cell penetration of Arg-rich CPPs.

Moreover, a highly conserved sequence (LRLLR) was found by Marks et al. [59] in peptides that were spontaneously translocating across membranes. This peptidic motif was later modified by making variants in the Arg positions and adding Trp to increase membrane binding, thereby resulting in sequences with increased translocation abilities [22]. These sequences were incorporated in CPPs in order to facilitate their direct permeability and elude endosomal entrapment.

The last important drawback of CPPs is their low cell, tissue, and organ selectivity. Attempts to overcome this problem were made by conjugating CPPs with a targeting moiety such as homing peptides or proteins, receptor ligands, or antibodies [22].

An additional possibility consists of designing peptides with both cell selectivity and cell penetration properties or in applying controlled delivery strategies [6]. Cell and tissue selectivity have been greatly enhanced in new generation peptides, in which CPPs are preferentially drawn to target a specific organ or tissue. For instance, crotamine, a cationic peptide that possesses anticancer, antimicrobial, and antifungal properties, delivers its cargos principally to actively proliferating cells such as cancer cells.

Another CPP that preferentially enters cancer cells is azurin, which exerts cytostatic and cytotoxic activity without affecting normal cells. Moreover, due to its affinity for brain endothelial cells, the rationally design peptide CB5005 was shown to penetrate into the brain and access cell nuclei, thereby emerging as a promising treatment for glioblastoma [25]. On the other hand, controlled delivery strategies can be applied to ensure a targeted delivery of CPP–cargo complexes. This involves activatable CPPs (ACPPs). ACPPs are generally polycationic CPPs whose adsorption and cellular uptake is minimized by an inhibitory domain, to which they are covalently attached by means of a trigger-responsive linker [4,60].

Compared to healthy tissue, tumorigenic tissue and its microenvironment are characterized by the overexpression of metalloproteases (MMP), acidic pH, and the upregulation of reactive oxygen species (ROS). ACPPs have mostly been developed for responding to these environmental triggers, thus becoming selectively activated only in the tumor-affected tissues [4]. The first ever described ACPPs comprised a polyanionic inhibitory domain connected to a cationic CPP through a MMP-sensitive PLGLAG linker that ensured its preferred uptake in MMP-rich sarcoma cells [61]. Since then, numerous advances have been made in the development of both ACPPs and their triggers. More specifically, CPPs have been developed that are inactivated by removable side chain modifications. In addition, approaches have been applied where conformational changes essential for CPP uptake were driven by external triggers [60].

## 7. Attempts to Deliver Drugs through the BBB

As mentioned earlier, the very low pinocytotic rate and the restricted paracellular transport, due to the presence of TJ between endothelial cells of brain capillaries, together with the presence of degrading enzymes and efflux pumps, make the BBB a selective barrier and the main impediment to deliver therapeutics to the CNS [4,9,10]. However, the ability of certain CPPs to cross biological barriers and deliver cargos into the cells, without disrupting the integrity of the membranes, has attracted much attention and raised the possibility that such peptides might be valuable agents to deliver biological drugs across the BBB. The first in vivo evidence of the CPP-driven release of proteins into the CNS was provided in 1999 by Schwarze and colleagues [62], who proved that, once intraperitoneally injected into mice, the TAT-β-galactosidase fusion protein was able to reach the brain. However, TAT is not the only CPP capable of delivering proteins across the BBB, but also RDP, a peptide developed by Fu et al. [63] starting from rabies virus glycoprotein, and a FGF4-derived peptide [64] were reported to have this ability.

Because CPPs are able to form nanoparticles when complexed with nucleic acids through electrostatic interactions, they have also been proposed as non-viral vectors for gene delivery and RNA interference in the CNS. Successful delivery to the nervous tissue is again one of the major challenges that gene therapeutics have to face, not only because of the impediment of the BBB but also because, due to their post mitotic nature and complex structure, neurons are particularly difficult to transfect. As of today, viral vectors are the most efficient carriers to release nucleic acid-based therapeutics. Among others, neurotropic viruses such as Herpes Simplex Virus-1 and Adeno-Associated Virus-9 seem to be the most appropriate to mediate the delivery to the CNS [5]. However, the clinical use of viral vectors can be limited by various factors including the complexity of large scale production and the generation of immune or inflammatory responses [5,21]. In a pioneering study, Kumar et al. [65] demonstrated that a chimeric peptide, formed by oligoarginine (9R) and a short peptide derived from rabies virus glycoprotein (RVG), namely, RVG-9R, could effectively deliver siRNAs across the BBB, determining a therapeutic effect after intravenous injection in mice with fatal viral encephalitis. Later, RVG-modified exosomes were shown to be capable of delivering siRNAs to the mouse brain after systemic injection [66]. Other CPPs were also proven to be able to deliver nucleic acid to the CNS including penetratin, which was shown to deliver siRNAs to the subventricular zone in adult mice [67].

Clinical studies in which CPPs were conjugated to chemotherapeutic drugs (e.g., Angiopep [68]) or were used as the main therapeutic agents themselves (e.g., p28 [69,70]) for the treatment of CNS tumors also demonstrated that CPPs can reach the CNS and deliver potential cargos after systemic administration in humans. Altogether, this amount of evidence suggests that CPPs can be considered as useful tools to ensure the access of therapeutics to the CNS parenchyma in pathological conditions.

In all the cases listed above, the therapeutic complex was administered systemically by intravenous injection/infusion, thereby requiring patient hospitalization and the support of specialized personnel. Not only does this procedure imply high costs, but, given the low selectivity of most CPPs, could result in the distribution of the CPP–drug complex to tissues other than the CNS, causing secondary effects. This has raised the question of exploring alternative routes of administration such as the intranasal route.

## 8. Nose-to-Brain Delivery

Traditionally, the IN route of administration has been used for the topical delivery of drugs for the treatment of local diseases such as rhinitis, allergies, or congestion. However, thanks to the efficient adsorption and permeability of the nasal mucosa to various molecules, this route of administration was also later explored for the systemic and brain distribution of therapeutics [23,71]. The N2B delivery, first developed by Frey in the late 1980s to target neurotrophic factors to the CNS [72], represents a minimally invasive method of bypassing the BBB and the cerebrospinal fluid (CSF) to release therapeutics to the brain and spinal cord while ensuring proper patient compliance and the potential for self-medication [23,71]. The IN delivery allows drugs, which would not otherwise cross the BBB, to reach the CNS within minutes. In addition, it eliminates the need for the systemic administration of therapeutics that are able to cross the BBB, thus ensuring a reduction in the side effects [18,73].

## 9. Transport of Therapeutics from Nose-to-Brain

IN administered therapeutics are delivered to the CNS through the olfactory and trigeminal neural pathways. In order to reach the CNS, they first need to cross the epithelial barriers in the nasal compartment, being transported from the nasal mucosa to the olfactory bulb (OB) or the brain stem, the entry points of the olfactory and trigeminal nerves, respectively. From these initial entry sites, they can disperse to other areas of the brain to reach their sites of action within the CNS [9].

Once in the nasal compartment, therapeutics need to avoid mucociliary clearance and penetrate the nasal mucus. Motile cilia, which cover most of the nasal cavity, are essential to promote the flow of the mucus toward the nasopharynx and thus normally exploit a protective action by removing inhaled substances that could damage the respiratory tract [74]. However, in the specific case of IN administrated drugs, mucocillary clearance is an impediment as it usually removes most of the therapeutics before they can be absorbed by the nasal mucosa, particularly when drugs exhibit low permeability capacities [23]. It follows that the development of effective IN drug formulations requires a careful consideration of their physico–chemical characteristics as well as of the cytoarchitecture and mucociliary clearance of the nasal mucosa [71].

Another protective layer that drugs need to cross in order to reach the nasal epithelium is represented by the mucus. The permeability of the mucus depends on its thickness and consistency as well as on the properties of the therapeutics themselves such as their size and charge [71].

**Figure 3:**
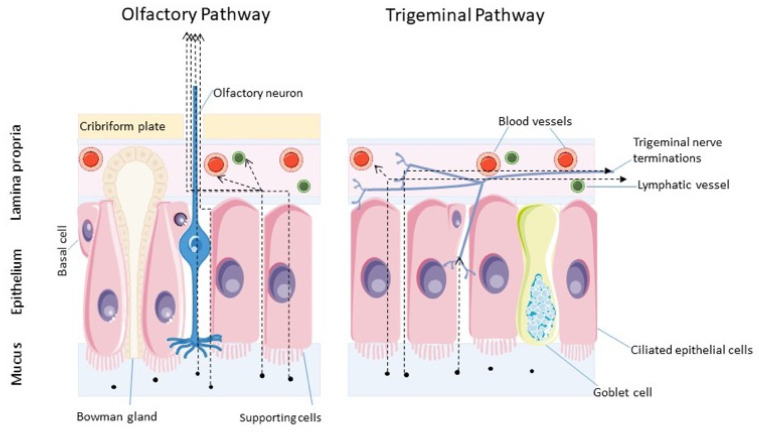
Schematic representation of the different routes described for N2B delivery. Upon administration in the nasal cavity, a drug (black dots) can reach the CNS via (i) an intracellular pathway, being endocytosed by either a neuron or a supporting cell and then shuttled to the CNS, or (ii) an extracellular pathway, travelling through the space between the olfactory neuron axons and the supporting cells or between two supporting cells. Once the nasal epithelium is crossed and the lamina propria is reached, drugs can also enter the blood or lymphatic vessels and join the systemic circulation, reaching peripheral organs.

Two possible mechanisms have been investigated for the passage of drugs from the nasal mucosa to the entry points in the CNS: the intracellular and the extracellular pathways (Figure 3).

The intracellular pathway starts with drug endocytosis into the olfactory or trigeminal axons, and continues with the intraneuronal transport within olfactory sensory neurons or trigeminal ganglion cells until a synaptic cleft is reached in the OB or the brain stem (Figure 3). At this level, release via exocytosis can take place [75]. Intracellular axonal transport is extremely slow. It has been reported to take up to 6 h for a substance transported along the olfactory nerve and up to 56 h to reach the brain stem via the longer trigeminal nerve [71]. However, numerous studies have reported a rapid N2B delivery within minutes, indicating that the intracellular pathway is unlikely to be the primary route [76,77,78].

On the other hand, the extracellular pathway, which is believed to deliver drugs to the brain in a short timeframe, begins with the penetration across the nasal epithelial cell layer to reach the lamina propria [74]. The nasal epithelium, despite being characterized by the presence of TJ between the different apical cells, displays a rather good permeability [71]. The regular cell turnover, typical of this tissue, is indeed regarded as responsible for the continuous rearrangement and loosening of the TJ between apical cells, which may facilitate the passage of large molecules [9]. Once the drug reaches the extracellular environment of the lamina propria, its arrival to the CNS remains uncertain. Therapeutics may be absorbed by the blood vessels, thereby entering the systemic circulation, or they can reach the lymphatic vessels and be transported to the deep cervical lymph nodes of the neck. Drugs absorbed into the systemic circulation may reach the CNS, but they need to cross the BBB or blood–CSF barriers [71,75,79]. It is also possible that, instead of being distributed throughout the systemic circulation, drugs enter the venous blood supply in the nasal compartment. Then, they can be promptly transferred through the carotid arterial blood flow to the brain and spinal cord [80].

If the therapeutic molecule continues its travel to the CNS through the extracellular pathway, it can either diffuse into the cleft between the axons and the supporting cells, which is filled with CSF and connect the subarachnoid space to the lamina propria, or it can move through or along the supporting cells. In the latter case, the active molecule can be endocytosed by supporting cells or travel through the intercellular spaces characterized by the presence of TJ. To migrate to the CNS via the extracellular pathway, the drug is thought to diffuse by a mixture of bulk flow, pulsatile pressures created by concurrent arterioles, or Brownian movements [71,81]. Translocation through the perineural space is much faster than intracellular translocation, and, given its shorter length, the olfactory neural pathway seems to be more feasible than the trigeminal pathway [9] (Figure 3).

Once the initial entry points in the CNS (i.e., the OB and the brainstem, via the olfactory and trigeminal neural pathways) are reached, drug molecules need to distribute from the nerve origins throughout the brain to reach their site of action and to produce a therapeutic effect. This final distribution may take place by means of intracellular or extracellular transport. The intracellular pathway consists of the transfer of the drug to second-order neurons synapsing with olfactory sensory neurons or trigeminal ganglion cells. The extracellular pathway, regarded as the main mechanism, consists of bulk flow within perivascular spaces of cerebral blood vessels [9,75].

## 10. Delivering Biotherapeutics through the Intranasal Route of Administration

Despite the advantages provided by the N2B route of administration for targeting of the CNS including the possibility of bypassing the BBB, only a few nasal products have been marketed for the treatment of CNS pathologies [79,82] (Table 2).

In the specific case of biotherapeutics, N2B delivery has proven to be effective in preclinical trials for the release of various peptides and proteins to the CNS. In particular, the N2B delivery of insulin, a protein regulating energy homeostasis, learning and memory acquisition [93], has been extensively studied. Brabazon et al. [94] traced the delivery of iodinated insulin in a rat model of traumatic brain injury, and 45 min after IN administration, detected the presence of [^125^I]-insulin into the OB, the area that displayed the highest concentration. The next highest levels were in the cerebellum, brain stem, hippocampus, and cortex, respectively. IN insulin was also shown to improve cognitive and motor function, reducing hippocampal lesion/edema, while leaving the blood levels of glucose unaltered. Using fluorescently-labeled insulin, Renner et al. [95] confirmed that insulin was able to travel along the olfactory nerves, thus reaching the OB. More recently, the results obtained by Lochhead et al. [93] suggest that insulin can also reach the brain, diffusing along the perineural spaces of the trigeminal nerve and distributing along the cerebral perivascular spaces. Finally, the pharmacokinetic profiles of IN and subcutaneous insulin were also compared, which revealed that, despite the absence of a significant difference in the mean brain concentration between the two routes of administration, the IN formulation displayed a 2000-fold increase in the brain:plasma area under the curve (AUC) ratio compared to the subcutaneous formulation, meaning that it is more suitable for a targeted delivery [96].

The glucagon-like peptide 1 (GLP-1) antagonist exendin (9–39) was also analyzed after IN administration for its effect on cognition and neuronal survival. Noteworthy, its iodinated form was shown to be delivered to the OB, anterior brain, hippocampus, cerebellum, and brain stem. Banks et al. [97] compared the blood and brain concentrations of the biotherapeutic after IN and intravenous (IV) administration, showing that the IN route resulted in a higher brain/serum ratio when compared to the IV route.

In accordance with the results obtained with insulin and exendin (9–39), Dhuria et al. [76] compared the pharmacokinetics in blood, CNS, and peripheral tissue of the neuropeptide hypocretin-1 after IN and IV administration in rats. Once again, IN administration emerged as the preferable route to obtain a targeted delivery of biologics to the CNS. Hypocretin-1 was indeed shown to reach multiple brain regions including the trigeminal nerve, the OB, and the cervical lymph nodes when administered via the IN route compared to an equivalent IV dose. At variance with this, IN administration resulted in significantly less delivery to the blood and peripheral tissues.

The IN delivery of the obesity-related hormone leptin has also been extensively addressed. Although leptin can normally cross the BBB, its transport is impaired in obesity, causing leptin resistance [98]. Because of its ability to bypass the BBB, IN delivery may contribute to solving this problem, providing an effective release of leptin to the CNS. A pharmacokinetic study performed by Fliedner et al. [99] on male Wistar rats showed that upon IN administration, about 81% of iodinated leptin ([^125^I]-leptin) was successfully delivered to the CNS within 30 min, with the highest levels in the hypothalamus and OB. IN leptin was shown to reduce appetite and induce weight loss in rats with diet induced obesity (DIO) to the same extent as in lean rats [100]. The IN route was also proven to be more effective than the intraperitoneal route for the delivery of leptin to the CNS. IN leptin was indeed reported to determine a reduction in terms of weight loss and food intake in DIO mice, while the intraperitoneal formulation showed no effects. Finally, IN leptin proved to be useful in relieving the sleep-disordered breathing associated with obesity [101].

It is important to note that the olfactory system of rodents displays some substantial anatomical and physiological differences when compared to humans including discrepancies in the nasal cavity volume and shape. It follows that the results obtained in humans may differ from the expected ones, based on preclinical evidence in rodents [9,102]. However, the N2B route has also been proven to be effective in delivering interferon-β (IFN-β) in rats [103,104] as well as in adult cynomolgus monkeys [78], whose nasal compartment exhibits a similar morphology to that of humans. In particular, the study conducted by Thorne et al., in accordance with previously obtained results in rats, underlined the ability of [^125^I]-IFN-β1b to enter the primate CNS along the olfactory and trigeminal routes [78].

Some promising results have been obtained in clinical trials testing IN formulations of various biotherapeutics including arginine-vasopressin [105], angiotensin II [106], insulin [107,108], and oxytocin [109]. These investigations proved that biological drugs can reach several areas of the human brain and exert pharmacological effects following the N2B route of administration. For instance, IN-administered insulin has been proven to be effective in reducing N1 and P3 amplitudes of auditory evoked potentials without modifying the blood levels of glucose in healthy subjects. This confirms that insulin can reach the brain directly, eluding the bloodstream [107]. Moreover, in accordance with the results obtained with anti-diabetic drugs in animal models of Alzheimer’s disease (AD), IN insulin showed the ability to modulate the plasma levels of amyloid β (Aβ) [110], thereby improving delayed memory and preserving cognitive function in patients with amnestic mild cognitive impairment and AD [111].

## 11. Challenges and Strategies to Achieve Efficient Intranasal Delivery

Despite the numerous advantages of N2B delivery, some limitations have also emerged with regard to this procedure. Among others, one important issue relates to the small dimensions of the nasal cavity that enable only the administration of small volumes of the therapeutic (maximum 150–200 µL in humans), making this route of administration suitable for highly potent drugs [71,73]. However, the exact dosing of intranasally administered drugs remains a challenge, especially considering that the anatomy of the nasal cavity may vary significantly between individuals [71]. Furthermore, to achieve a meaningful therapeutic effect, it is extremely important to deliver the highest dose precisely to the olfactory region, which is complex to realize by using traditional spray pumps. In addition, because patients self-administer the drug, the efficacy of the treatment may also be affected by some inaccuracy in the administration technique [81,102]. These problems have been partially resolved by the development of new devices such as Vianase™ and Intravail™ [112], which enable a more predictable and controlled intranasal drug delivery than typical nasal spray bottles.

Protective barriers in the nasal mucosa are the main reasons why intranasally administered substances display a reduced bioavailability, with only about 1% of the administered dose reaching the brain [80]. The mucus coating, which protects the underneath nasal epithelium, is the first barrier that the drug must cross to access the CNS. It is composed primarily of mucins, kept together by electrostatic interactions, which present a net negative charge. Hydrophobic and charged hydrophilic molecules as well as drugs larger than 0.5 kDa in size have been shown to diffuse poorly in the mucus and to be prone to elimination by mucocillary clearance mechanisms, reducing the contact time necessary for drug absorption.

On the other hand, small, uncharged, hydrophilic molecules can move quickly through the mucus and the matrix of mucins. Normally, molecules with a size smaller than 0.4 kDa can freely diffuse and penetrate through the nasal epithelia [73,81]. In addition, the presence in the nasal mucosa of both efflux transporters such as P-glycoprotein and multidrug resistance associated proteins as well as drug metabolizing enzymes including cytochrome P450 and proteases reduces the absorption of the drugs, thus limiting the efficiency of IN delivery to the brain [80,81]. Moreover, the drug absorption by the nasal vasculature and the presence of TJ in the nasal epithelium may also represent limiting factors to the direct delivery to the brain [80].

Because of (i) the reduced brain bioavailability of IN applied drugs, (ii) the limited volume of drug that can be applied intranasally, and (iii) the need to avoid irritation of the nasal compartment, additional strategies need to be established in order to ensure an effective and safe delivery of neurotherapeutics through the IN route. Attempts to overcome the limiting barriers above-mentioned have been made by developing novel formulations to improve drug solubility, increase permeability across the nasal epithelium, and reduce the clearance from the nasal compartment [80].

To achieve an adequate absorption and bioavailability in the CNS after IN administration, a therapeutic must have sufficient solubility at the site of delivery (i.e., the nasal epithelium) [80]. Drugs can be administered in different forms including dry powders, which are highly stable, and liquid suspensions, which are the most common nasal preparations in the specific case of drugs with low aqueous solubility. In both cases, the slow dissolution of poorly soluble drugs represents an important limiting factor because most of the therapeutic, which is unable to penetrate the mucus layer, tends to be cleared toward the pharynx [73]. To solve this issue, therapeutics can be solubilized by encapsulation in carriers such as cyclodextrins, microemulsion formulations, or nanoparticles [80,102] (Table 3). In particular, cyclodextrins consist of a hydrophilic shell and a hydrophobic cavity, where the poorly water-soluble drug can accommodate. They can basically form inclusion complexes with many substances, thus increasing the solubility and brain uptake after IN administration. For instance, IN delivery to the brain of Galanin-like peptide (GALP) was increased approximately threefold upon complexation with cyclodextrins [113]. Recently, β-cyclodextrin was also proven to be useful for the IN delivery to the mouse brain of allopregnanolone, a steroid with anti-seizure activity, thereby elevating the seizure threshold [114].

Microemulsion and nanoemulsion formulations have been reported to improve the direct transport to the CNS of small molecules such as clonazepam, sumatriptan, risperidone, zolmitriptan, and nimodipine [80]. Furthermore, an emulsion-like formulation was patented for use with hydrophobic proteins and/or peptides that are either water-insoluble or susceptible to precipitation at physiological pH. These include, for example, growth differentiation factor 5 (GDF5) [115]. The use of this emulsion-like formulation for the IN administration of GDF5 improved delivery to all regions of the CNS and to the trigeminal nerve [80] (Table 3). Finally, other nanometric drug carriers including polymeric or lipid-based nanoparticles were taken into consideration when it was necessary to improve the access to the CNS by therapeutics. Nanoparticles have the advantage of protecting the drug from enzymatic and chemical degradation, and they are suitable for functionalization with permeability-enhancing components or transporter inhibitors [73].

Another strategy that has been applied to maximize drug absorption at the nasal cavity, potentially enhancing the delivery to the CNS along the olfactory and trigeminal pathways, consists in reducing the clearance, thereby prolonging the residence time of the formulation at the delivery site. To achieve this result, mucoadhesive and viscosity enhancing agents have been employed including the cationic agents chitosan, carboxymethylcellulose, lectin, and polyacrylic acid [80,102]. For instance, the copolymer chitosan, which is positively charged, can form electrostatic interactions with the negatively charged mucins, thus increasing the time available to the drug for absorption. It is noteworthy that chitosan is biocompatible, biodegradable, and displays a low toxicity, as demonstrated by the fact that it is already used as a supplement to weight loss products. Moreover, aside from its mucoadhesive properties, it is also thought to enhance the penetration across cellular membranes [71,80,102]. Khan et al. [116] reported both a significant increase in the retention time as well as the enhanced cell permeability of buspirone in the rat nasal compartment when formulated with 1% chitosan. Furthermore, they showed that the addition of 1% chitosan and 5% hydroxypropyl β-cyclodextrin could enhance the N2B delivery of buspirone, resulting in twofold higher brain AUC for the mucoadhesive formulation when compared to the simple buspirone solution [117]. Moreover, both reversible and irreversible ciliostatics and ciliotoxic drugs such as chlorbutol and hydroxybenzoates can be used to further slow mucocillary clearance and to increase the drug resistance time in the nasal compartment [81] (Table 3).

Biogels (i.e., systems that can modulate their viscosity in response to physical or chemical stimuli [81]) have also been used to increase the retention time and drug absorption at the nasal compartment. Many polymers can act as biogels including poloxamer, polyacrylic acid derivatives, or cellulose, and their use seems to be quite promising [81]. For instance, Dalvi et al. [118] recently found that the brain AUC of the anti-epileptic rufinamide was significantly higher when administered in xyloglucan-based, heat triggered biogels when compared to the simple suspension upon IN application [512.17 vs. 104.28 min × (µg/g)] (Table 3). In addition, other studies have reported significant improvements in brain bioavailability with different kinds of biogels including pluronic acid and Carbopol^®^ gels for rivastigmine tartarate [122], poloxamer gels for rasaligine [123], and mucoadhesive thermoreversible Lutrol F127 gels for venlafaxine hydrochloride [124].

Another valid approach to increase the drug residence time at the delivery site is to reduce the clearance from the nasal cavity due to efflux transporters or absorption by the nasal vasculature (Table 3). Efflux transporters such as P-glycoprotein are known to reduce the brain uptake of therapeutics upon intranasal administration. However, the use of appropriate transporter inhibitors such as rifampin has been proven effective in overcoming this limitation, resulting in a greater brain uptake of the drugs [119,120]. On the other hand, vasoconstrictors have been used to ensure a minor drug clearance into the bloodstream. Dhuria et al. [121] found that the addition of the vasoconstrictor phenylephrine to nasal formulations of the neuropeptide hypocretin-1 or the dipeptide L-Tyr-D Arg resulted in both a reduction in the amount of drug adsorbed into the blood [65% reduction for hypocretin-1 and 56% reduction for the dipeptide] and a 3-fold increase for both in the amount delivered to the OB (Table 3).

However, increasing the permanence into the nasal cavity can also prolong the interaction of the drugs with degrading enzymes and proteases, thus resulting in enhanced loss of the therapeutic. To overcome this impediment, the use of enzyme inhibitors such as P-glycoprotein inhibitors, CYP450 inhibitors, and acetazolamide has been tested with positive results. One point that should be considered is that increasing the exposure to the drug may also lead to hypersensitivity, thus reducing the tolerability to the medicament [81].

To achieve efficient delivery to the CNS through IN administration, drugs need to have sufficient solubility at the site of administration as well as to efficiently permeate the nasal epithelia, in order to be subsequently transported via extracellular mechanisms along the olfactory and trigeminal nerves. When their size and/or polarity interfere with the passage through the membranes, the addition of a permeation enhancer in their formulation may improve the delivery to the nervous system. However, permeation enhancers (e.g., surfactants, bile salts, lipids, cyclodextrins, polymers) and TJ modifiers, which are especially useful for the delivery of hydrophilic compounds and macromolecules, are accompanied by nasal toxicity due to disruption of the nasal epithelia [80,81,102]. Thus, the development of alternative approaches to ameliorate the CNS uptake of IN therapeutics is highly desirable. In this context, CPPs have been proposed as permeation agents, both individually or in combination with polymers and/or lipids, to create more complex nanosystems.

## 12. CPPs to Improve N2B Delivery of Biologics

Due to their large size, hydrophilic nature, and poor penetration capacity, biotherapeutics struggle to permeate the nasal epithelium and reach the CNS after IN administration [125]. For example, results obtained by Kamei and Takeda-Morishita [20] on the model peptide insulin revealed that IN-administered peptidic drugs require 10-fold higher doses to achieve levels in the brain that are similar to IV-administered biotherapeutics. However, achieving such high doses in the nasal compartment may represent a problem, mainly because of the small volume that can be administered intranasally. To maximize the advantage of the IN route of administration in delivering therapeutics to the brain, new strategies have been developed including the use of CPPs as carriers and permeation agents for biologics.

When achieved with traditional permeant agents, the enhanced permeability of the nasal mucosa can lead to irritation and disruption of the epithelia integrity (e.g., upon chronic treatments) [80,81,102]. Conversely, CPPs have been proven to be capable of penetrating biological membranes without damaging them [4]. Several toxicity studies have been conducted on the nasal mucosa to assess the integrity of the epithelium and OB, the morphology and integrity of the cilia, and the inflammatory status. These revealed that no significant toxic responses were generated upon the IN administration of CPP-containing formulations [126,127,128,129,130]. For instance, the safety of L-penetratin and the penetratin analog “PenetraMax”, used as permeating agents for IN delivery, was assessed in a one-month subchronic toxicity study. This proved that neither peptides determined local or systemic toxicity upon long-term IN application [128].

Furthermore, CPPs have been reported to enhance the N2B delivery of biodrugs including insulin [20,131,132], exendin-4 [133], glucagon-like peptide-2 (GLP-2) [134,135], leptin [136], neuromedin-U [137], and human acidic fibroblast growth factor (haFGF) [127,138,139] (Table 4). In particular, IN insulin has been proposed as a pharmacological agent for the treatment of AD, although its pharmacokinetic profile is unfavorable. This implies that the dose needed to reach a sufficient amount of biologic in the brain is much higher than the one required upon intravenous administration [20]. To overcome this issue, Kamei et al. [20] proposed exploiting penetratin as a delivery agent for IN insulin delivery. They first investigated the effects of non-covalently conjugated L- and D-penetratin on direct brain transport as well as on the systemic absorption of insulin after IN administration. They discovered that both CPPs increased insulin levels in the OB and the whole brain. L-penetratin resulted in being the most effective of the two peptides, while D-penetratin emerged as the most efficient in maintaining a favorable AUC_brain_/AUC_blood_ ratio and therefore in determining less systemic exposure (Table 4). The same group later performed a quantitative assessment of the brain distribution of non-covalent insulin-(L/D)-penetratin complexes upon IN administration, confirming that conjugation with both L- and D-penetratin resulted in an increased insulin accumulation in the brain. In particular, the signal of a radioactive form of insulin (^64^Cu-NODAGA−insulin) was increased by the conjugation with L- or D-penetratin, with the greater signal obtained in the OB with L-penetratin. To confirm the results obtained with radioactivity, the concentration of IN administered unlabeled insulin was assessed by ELISA in six different brain compartments (i.e., OB, hypothalamus, hippocampus, cerebral cortex, cerebellum, and brain stem as well as in the plasma and CSF). Once again, the concentration of insulin was higher in the OB with both L- or D-penetratin. Moreover, in the case of L-penetratin, insulin concentration that resulted also increased in the hypothalamus, cerebral cortex, cerebellum, and brain stem. Unfortunately, the plasma concentration of the unlabeled insulin was significantly increased with L-penetratin, with the AUC ratio in the whole brain and plasma closely similar to that achieved with intravenous administration (0.068 for IN administration and 0.15 for IV administration), thus opening up to possible undesired systemic effects [132] (Table 4). They further assessed whether the use of penetratin as a delivery agent could increase the pharmacological efficacy of insulin in the treatment of dementia. More specifically, Kamei et al. [131] examined the therapeutic effect of IN insulin with or without L- or D-penetratin on cognitive dysfunction on the SAMP8 mouse model of dementia at two different stages of senescence. Remarkably, L-penetratin resulted in being effective in enhancing the therapeutic efficacy of insulin, improving mild cognitive dysfunction during the early stage of dementia in the absence of Aβ accumulation (Table 4). Conversely, it was ineffective in recovering the severe cognitive dysfunction that is typical of the advanced stage of the disease, which involves Aβ accumulation in the brain. Interestingly, chronic IN treatment with insulin alone was shown to contrast hippocampal degeneration in aged SAMP8 mice, while co-administration with L-penetratin was reported to further accelerate the formation of Aβ plaques in the hippocampus. Moreover, L-penetratin was shown to enhance the amount of insulin that reached the systemic circulation, resulting in the reduction in plasma glucose. From the results by Kamei et al. [20,131,132], we can conclude that penetratin enhances the N2B delivery of insulin. In the specific case of early stage dementia, it is also effective in ameliorating the therapeutic action of insulin, even though it causes a parallel and undesirable hypoglycemic effect (Table 4). This has raised the question of developing additional strategies to boost the direct transport of drugs to the brain without enhancing systemic absorption, in order to avoid unwanted systemic activities by neurotherapeutics.

In an attempt to treat severe cognitive dysfunction during the advanced stage of dementia, the same research group analyzed the effects of non-covalent CPP conjugation on N2B delivery of the GLP-1 receptor agonist exendin-4 [133]. In particular, co-administration with L-penetratin was shown to facilitate the transport of exendin-4 from the nose to the OB and to increase exendin-4 concentration in the hypothalamus, hippocampus, cerebral cortex, cerebellum, and brain stem, resulting in a whole brain concentration of 1.71 ± 0.33 ng/g tissue compared to 0.74 ± 0.10 ng/g tissue for the control group. GLP-1 receptor agonists including exendin-4 promote the activation of insulin signaling in the brain through GLP-1 receptor stimulation. Hence, they were believed to correct the severe condition associated with AD and were characterized by Aβ oligomer-dependent depletion of the insulin receptor [140]. Preliminary results revealed that co-administration of exendin-4 and L-penetratin was insufficient to facilitate insulin signaling in the brain [133]. Thus, the therapeutic effects of IN exendin-4 were tested in the presence or absence of insulin. The results of these experiments showed that N2B delivery of exendin-4, co-administrated with L-penetratin and supplemented with a low dose of insulin, improved cognitive and spatial functions in the SAMP8 mouse model of dementia (Table 4). This study also confirmed that the co-administration of exendin-4 and insulin with L-penetratin resulted in an increase in the blood concentrations of both peptides. However, in this case, due to the low dose of insulin (8 IU/mL compared to 30 IU/mL of the previous study), blood glucose was maintained to safe levels [133].

Another biotherapeutic that has been proposed for the treatment of AD is the neurotrophin-like growth factor haFGF. An IN formulation of haFGF has been developed by fusing haFGF to the CPP TAT (TAT-haFGF) in order to boost haFGF delivery to the brain [138]. Lou et al. [138] first assessed whether the covalent conjugation with TAT could enhance the access of haFGF to the nervous system. This was achieved by comparing the concentration of the growth factor in the brain of mice treated with either IN TAT-haFGF or unconjugated haFGF at different time points. ELISA results proved that the conjugation with TAT significantly enhanced the delivery and distribution of the biotherapeutic to the brain at all time points analyzed (5, 15, 30, 60, 120 min). Moreover, in the serum, the levels of haFGF remained 10-fold lower than in the brain, despite the small non-significant increase determined by the conjugation with TAT (Table 4). This evidence proves that the IN route can provide targeted delivery to the brain. The same group [138] also tested the therapeutic efficacy of TAT-haFGF on the SAMP8 model of dementia, and found that the IN treatment with TAT-haFGF was effective in correcting cholinergic deficits. Furthermore, it improved the learning and memory abilities in SAMP8 mice by regulating the activity of the acetylcholinesterase (AchE) and choline acetyltransferase (ChAT) enzymes, which are responsible for the metabolism of the neurotransmitter acetylcholine (Ach).

Treatment with TAT-haFGF was also shown to decrease the amount and size of Aβ deposits in the hippocampus and cortex of SAMP8 mice as well as significantly decrease the number of apoptotic neurons. Furthermore, it alleviated the oxidative stress by regulating the activity of relevant enzymes, thereby ameliorating the neuronal microenvironment (Table 4). Altogether, these small therapeutic effects, achieved at the molecular level, resulted in the improvement in the learning and memory abilities of SAMP8 mice. The same research team later tested the safety and brain-related tissue distribution characteristics of the biotherapeutic TAT-haFGF in Sprague-Dawley rats [127]. They found no significant difference in the morphology and integrity of the cilia in the toad palate as well as in the structure or number of olfactory sensory neurons between the groups treated with the vehicle or TAT-haFGF. Thus, they concluded that the biotherapeutic was safe and caused no toxicity in the nasal compartment. Tissue distribution was assessed using the radioactive form of the active molecule ^125^I-TAT-HaFGF. At 15 min post-administration, ^125^I-TAT-HaFGF was detected in the hypophysis, OB, brainstem, and cerebellum. At 30 min after IN administration, radioactivity persisted in the brainstem, cerebrum, and cervical spinal cord, while after 1 h, the signal in these areas weakened. The peak of radioactivity in the whole brain, OB, brainstem, cerebellum, and cervical spinal cord was reached at 30 min after IN administration and decreased after 1 h. Distribution of the active molecule was consistent with the involvement of the olfactory and trigeminal pathways of delivery. The radioactive molecule could also be found in the blood 15 min after IN administration, demonstrating rapid nasal absorption into the systemic circulation. The same research group also assessed differences in the TAT-haFGF pharmacokinetic and pharmacodynamic profiles between the IV and IN administration routes in the AβPP/PS1 mouse model of AD [139]. The IN administration route was found to be more effective in delivering the biotherapeutic to the brain, as demonstrated by the fact that the TAT-haFGF levels were significantly higher in the mouse hippocampus, cortex, and OB than those achieved with the IV route at the same dose. Noteworthy, the levels in the systemic circulation remained significantly lower. The ability of the IN administration to achieve higher levels of TAT-haFGF in the brain resulted in a more significant improvement in cognitive functions than the one achieved with IV injections of the same dose. IN delivery of TAT-haFGF was indeed shown to be more effective in both increasing the ACh levels in the mouse brain and reducing the number and size of Aβ plaques in a dose-dependent manner when compared to IV delivery (Table 4).

As mentioned earlier, the IN delivery of the obesity-related hormone leptin has attracted great interest nowadays as it may provide an option to solve leptin resistance, a phenomenon likely due to the impaired transport of leptin across the BBB [98]. Khafagy et al. [136] recently proposed the non-covalently bound L-penetratin as a suitable delivery agent to enhance the N2B delivery of leptin. L-penetratin was shown to promote the systemic absorption of leptin with a nearly 10-fold increase and to stimulate brain distribution with a nearly 6-fold increase in the OB. After a single intranasal application, the concentration of leptin was found to be particularly elevated in the anterior part of the brain close to the administration site (i.e., the OB), and in the hypothalamus, the region located parallel to the trigeminal nerve. The systemic and brain distribution of leptin was also assessed after long-term repeated IN administration of the mixed solution of L-penetratin and leptin. The plasma concentration of leptin resulted in being significantly increased 15 min post-administration, particularly at the late stage of the long-term study. In contrast with this, the concentration was very weakly enhanced at the early stage.

The long-term co-administration with L-penetratin was confirmed to increase leptin levels in the OB and hypothalamus, the latter being the target of leptin treatment of obesity. However, the absolute concentration was lower than that observed after a single administration. Khafagy and colleagues [136] also examined the therapeutic effects of the IN administration of the mixed solution of leptin and L-penetratin in the long-term study. They found that the continuous delivery of leptin induced appetite suppression, which was mediated by the stimulation of leptin receptors and activation of the downstream effector *Signal transducer and activator of transcription 3* (Stat3), with a consequent reduction in plasma triglycerides and, eventually, decreased body weight gain (Table 4).

The ability to promote N2B delivery of the new generation CPP LMWP has also been evaluated. LMWP is characterized by a compact region of positively charged Arg residues and was shown to facilitate the transduction of peptides and proteins into live cells [25], to promote the delivery of siRNAs into cancer cells [141], and to enable the permeation of insulin through the intestinal epithelial cell membrane and mucosal layer [142]. The ability of LMWP to deliver biotherapeutics to the brain via the IN route has been tested by administrating a CY5 dye-labeled LMWP–bovine serum albumin (BSA) conjugate to BALB/c nude mice [125]. The LMWP–BSA complex exhibited the capability to reach the brain, with high delivery levels in the OB and moderate distribution to the rest of the cerebral tissue. In contrast, BSA alone was reported to be retained into the nasal cavity and was not distributed to the brain. Moreover, the covalent conjugation with LMWP was shown to ensure a deep inward penetration into the cerebral tissue, whereas the native protein hardly showed any diffusion (Table 4). In addition, both BSA and LMWP–BSA were not detected in the major organs, indicating that LMWP did not enhance intranasal absorption into the systemic circulation [125]. To assess whether a biologically active protein retains its activity after being intranasally delivered to the CNS, Lin et al. proceeded with the IN administration of LMWP-conjugated horseradish peroxidase (HRP) or β-galactosidase (β-gal), followed by the detection of their enzymatic activity in the brain. They found that administration of the proteins in conjunction with LMWP could enhance the retention of their enzymatic activity after N2B delivery [125] (Table 4).

The strategy proposed for boosting the IN delivery of the neuropeptide neuromedin-U (NMU) for the treatment of inflammation-mediated amnesia is slightly different from the ones described so far. In fact, not only does it involve the conjugation of NMU with the arginine-rich CPP R8, but also with a penetration-accelerating sequence (i.e., PAS or F4), which is added to promote endosomal escape, thus creating the two different NMU derivatives PAS-R8-NMU and F4-R8-NMU [137]. PAS-R8-NMU was found to be more stable and more efficiently delivered to the CNS upon IN administration than F4-R8-NMU (Table 4). Moreover, while both IN-administered NMU derivatives prevented or reduced LPS-induced memory impairment in mice, native NMU, PAS-R8, and F4-R8 did not, thus reinforcing the idea that conjugation with the CPP and the penetration-accelerating sequence is necessary for adequate NMU delivery [137] (Table 4).

A very similar strategy has been proposed for the N2B delivery of GLP-2 for the treatment of major depression. To ensure an effective delivery to the brain, GLP-2 was conjugated with both a CPP and a penetration-accelerating sequence, thereby obtaining the GLP-2 derivative PAS-CPP-GLP-2, in which the CPP portion consisted of R8. Sasaki-hamada et al. [134] demonstrated that the PAS portion of the GLP-2 derivative was essential for IN delivery and the therapeutic effect of the compound. PAS-CPP-GLP-2, but not CPP-GLP-2 or the tricyclic antidepressant imipramine used as controls, was shown to exert an antidepressant effect in adrenocorticotropic hormone (ACTH)-treated mice. Brain distribution of both fluorescein isothiocyanate (FITC)-labeled PAS-CPP-GLP-2 and CPP-GLP-2 was also assessed upon IN administration. While PAS-CPP-GLP-2 was found to reach the hippocampus, CPP-GLP-2 was unable to distribute throughout the brain. Moreover, the FITC-labeled PAS-CPP-GLP-2 group was found to exhibit a denser fluorescence in the hippocampus, dorsomedial hypothalamic nucleus, and rostral ventrolateral medulla when compared to the vehicle group. Later, Akita et al. [135] investigated whether the CPP portion of the GLP-2 derivative was essential for IN delivery to the brain. The therapeutic efficacy of GLP-2, CPP-GLP-2, PAS-GLP-2, or PAS-CPP-GLP-2 was tested upon IN administration in ACTH-treated mice through the forced swimming test (FST). In accordance with the previous observations by Sasaki-hamada et al. [134], only the PAS-CPP-GLP-2 molecule was capable of significantly reducing the immobility time, thereby showing an antidepressant-like effect (Table 4). Both the CPP-GLP-2 and PAS-GLP-2 derivatives did not produce a pharmacological effect, thereby suggesting that the CPP and PAS portions are essential to ensure the access to the brain of a therapeutically effective concentration of derivative upon IN administration. Furthermore, IN and i.c.v. administration of the same dose of PAS-CPP-GLP-2 resulted in being similarly effective, suggesting that the delivery efficacy of both routes of administration is comparable for this particular derivative. Finally, the systemic absorption of PAS-CPP-GLP-2 was extremely low, as demonstrated by the fact that the plasma concentration of the derivative was below the detection limit in the specific assay used to assess its levels.

## 13. CPP-Functionalized Nanocarriers for IN Delivery

Nanometric drug carriers can improve the N2B delivery of biodrugs due to their capacity to enhance the stability of encapsulated therapeutics against chemical and enzymatic degradation [143]. Despite different nanosystems being developed for IN delivery, most experimental evidence still arises from studies based on the delivery of small molecules, with a few examples relative to biodrugs.

A first instance of nanosized drug carriers for N2B delivery are polymeric carrier nanosystems. These consist of nanocarriers made of polymers that can be customized to adjust the surface charge, cargo-loading, and release properties. They include two types of structures (i.e., polymeric nanoparticles and polymeric micelles) [73]. Polymeric nanoparticles can be described as matrix-like compact colloidal systems consisting of water-insoluble polymers, with a size ranging from 1 to 1000 nm, which can be loaded with active compounds [73,144]. Advantages in the use of polymeric nanoparticles such as nanocapsules and nanospheres include their ability to protect cargo molecules against the environment while improving their bioavailability and therapeutic indices as well as their controlled drug release capacity [144]. Various polymers have been used to create this nanosystem subtype including the biocompatible and biodegradable copolymer poly(lactic-co-glycolic acid) (PLGA), which has been proven to be useful in delivering hydrophobic drugs within nanoparticles [73]. The surface of PLGA nanoparticles can be further coated or conjugated with compounds to enhance their delivery to target tissues. Among the different compounds, there is the copolymer chitosan, which can facilitate intranasal delivery due to its mucoadhesive and TJ-opening capacity. Various chitosan derivatives and lectins or other ligands specific to the nasal epithelium have been shown to increase the retention time in the nasal compartment [81]. For instance, chitosan-coated PLGA nanoparticles (CS-PLGA-NPs) have been developed and tested as nanocarriers for the IN administration of curcumin for the treatment of Alzheimer’s disease [145]. Nanoparticles made of poly(lactic acid) (PLA), a simpler polymer consisting of PLGA without polyglycolic acid monomers, have also been shown to be effective in improving IN transport [81]. For example, wheat germ agglutinin-conjugated poly(ethene glycol)-PLA (WGA-PEG-PLA) nanoparticles have been tested for the IN delivery of coumarin with promising results in terms of the absence of cilia toxicity and brain uptake [146].

The composition of polymeric micelles, the second subtype of polymeric carrier nanosystem, differs substantially from that of polymeric nanoparticles, being made of amphiphilic block copolymers that self-assemble. These include, for example, poly(ethylene glycol)-block-poly(D,L-lactide), which creates a nanosystem consisting of a hydrophobic core that can incorporate hydrophobic drugs, and a hydrophilic corona, which stabilizes the interface between the core and the external environment [73]. For instance, poly(ethylene glycol)-block-poly(D,L-lactide) micelles have been proposed as N2B delivery agents for the hydrophobic molecule baicalein, a flavonoid compound with antioxidant and anti-inflammatory properties as well as a possible neuroprotective feature, with promising results in terms of safety, brain maximum (peak) concentration (Cmax), and AUC [147].

Solid lipid nanoparticles represent another example of nanometric drug carriers proposed to enhance the N2B delivery of therapeutics. Due to their biocompatible and biodegradable lipid composition, they are believed to have a high safety profile, while maintaining high stability and improving the bioavailability of the cargo molecule. Moreover, lipid nanoparticles are thought to have greater stability upon storage and to be cheaper in mass production when compared to polymeric formulations. In addition, they guarantee the controlled release of the transported active molecule. However, the lipid composition must be extensively controlled, since the inclusion of some fatty acids (e.g., phosphatidylcholine, phosphatidylserine, and phosphatidylethylamine) may determine rapid clearance of these nanosystems from the nasal epithelia [73,81]. When properly formulated, solid lipid nanoparticles can increase nasal retention, thus enhancing brain delivery [73]. For instance, solid lipid nanoparticles have been tested as delivery agents for the antipsychotic drug risperidone upon IN administration. Patel et al. [148] found a 10-fold higher delivery to the brain of this formulation of risperidone when compared to the simple solution, thus confirming the usefulness of this nanosystem in N2B delivery.

The last few examples of nanometric drug carriers are represented by liposomes and liposome-related vesicular nanosystems. Liposomes are biocompatible and biodegradable drug delivery systems, composed of vesicles made of phospholipid and cholesterol bilayers, which can incorporate both hydrophilic or hydrophobic therapeutic agents that are entrapped in the aqueous core and in the hydrophobic region of the lipid bilayer, respectively. The ease by which these nanocarriers can be functionalized led to the development of several variants of these nanoparticles including transferosomes. The latter are characterized by high flexibility and deformability due to the incorporation of edge activators in their lipidic bilayers [10,73]. Liposomes have been actively investigated as tools to facilitate the N2B delivery of therapeutics for the treatment of various neurological and neurodegenerative diseases such as AD, Parkinson’s disease, and ischemic stroke [8]. These nanosized delivery agents have been shown to successfully reach the brain via the intranasal route [10]. For instance, the liposome-encapsulated β-sheet breaker peptide H102 was shown to be successfully delivered to the CNS via IN delivery and to reach a significantly increased AUC in the hippocampal region when compared to the solution alone, with no important toxic reactions [149].

However, the composition and surface properties are important factors to consider when trying to deliver a liposome formulation via the N2B route. Commonly used phospholipids for the formulation of liposomes include both synthetic lipids (e.g., 1,2-dipalmitoyl-sn-glycero-3-phosphocholine and ethyl-phosphatidylcholine) and natural lipids (e.g., phosphatidylcholine, sphingomyelin, and lecithin). In spite of the numerous advantages of using natural phospholipids including the fewer solvents used for their extraction and the greater ease with which they are accepted by regulatory authorities, it is important to note that they are more prone to contamination by viruses, prions, or toxins when compared to their synthetic counterparts. Another lipid molecule that plays an important part in maintaining the stability of the membranes in vitro and in vivo is cholesterol. However, it should be considered that cholesterol can also reduce the permeability and alter the function of the lipid bilayer [10]. Noteworthy, surface properties have also been shown to be crucial for determining the efficient delivery to the CNS through the intranasal route. A recent study assessed the effects of the liposome surface charge and functionalization with polyethylene glycol (PEG) on the kinetics and distribution in the brain and spinal cord upon IN administration [8]. Kurano et al. [8] used both ex vivo fluorescence imaging of fluorescently-labeled liposomes and radioactivity detection of [^3^H]-cholesterol-labelled liposomes to visualize the spatiotemporal distribution in the CNS of liposomes with different surface characteristics. The peak liposome distribution occurred at 90 min, while the same distribution was confirmed at 120 min post-administration. Positively-charged liposomes were found to migrate, especially to the OB and forebrain, thus following the olfactory route, while negatively charged liposomes were reported to migrate preferably to the hindbrain, bulbospinal tract, and trigeminal nerves. Neutral liposomes were shown not to distribute to the hindbrain and spinal cord, unless they were functionalized with PEG, upon which they widely distributed to the entire CNS. Due to their higher stability in the interstitial fluid of the perivascular and perineural cavities, neutral PEG-modified liposomes are thought to be more prone to diffusion in the nervous system when compared to charged liposomes. However, because they do not actively interact with tissue and cell membranes, they are internalized in concentrations that are insufficient to determine a therapeutic effect. To overcome this problem, surface functionalization with permeation enhancers has been proposed.

The size is a key factor that actively influences the N2B delivery of every type of nanosystem. Studying the IN delivery to the CNS of polymeric nanoparticles of different diameters ranging from 88 nm to 102 nm and 560 nm, loaded with Nile red dye and administrated to rats, Kubek et al. [150] found that only the smaller nanoparticles, with a mean diameter between 80 and 100 nm, could deliver their content to various areas of the CNS including the OB, septal nuclei, insular cortex, hippocampus, and thalamus. This result was later confirmed by Kanazawa et al. [151] by examining the biodistribution, following IN administration, of coumarin-loaded polymeric micelles in a range of diameters between 100 and 600 nm in rats. Once again, the higher concentration of cargo molecules in the brain was achieved with 100 nm micelles, further confirming that nanosystems of this size are the most promising for N2B delivery. Therefore, 100 nm was considered the ideal diameter for a nanosystem to efficiently deliver its content to the brain via the IN route.

Each of the previously described nanosystems is suitable for functionalization with CPPs, which may improve both penetration across the nasal mucosa and delivery to target cells. For instance, after determining the ideal diameter of their methoxy poly(ethylene glycol)/poly(ε-caprolactone) (MPEG-PCL) polymeric micelles, Kanazawa et al. [151] assessed whether functionalization with the CPP TAT could influence brain distribution upon IN administration. Coumarin-loaded MPEG-PCL-TAT micelles differed in size and charge when compared to the coumarin-loaded MPEG-PCL micelles without TAT, being smaller and positively charged. No significant difference could be spotted in the coumarin brain distribution 1 h after IN administration between the polymeric micelles functionalized with TAT and those lacking TAT. However, 4 h post-administration, coumarin concentration in the rat brain was nearly 5-fold higher when administered using MPEG-PCL-TAT micelles. Noteworthy, the brain distribution of coumarin by the MPEG-PCL micelles was lower at 4 h after administration when compared to 1 h post-administration, suggesting the difficulty of MPEG-PCL micelles in penetrating into brain cells. Conversely, in the case of the MPEG-PCL-TAT micelles, the coumarin distribution was higher at 4 h than at 1 h post-administration, confirming their ability to penetrate into cells. Finally, the concentration of coumarin in non-target tissues including the liver, lung, heart, kidney, and spleen, was lower in rats treated with the coumarin-loaded MPEG-PCL-TAT when compared to animals administered with the coumarin solution alone, suggesting that these polymeric micelles may be useful for brain targeting via IN administration (Table 5). The same research group compared the efficacy of MPEG-PCL and MPEG-PCL-TAT in delivering the anti-cancer drug camptothecin (CPT) through the N2B route for the treatment of intracranial cancer. The MPEG-PCL-TAT polymeric micelles were more effective than the MPEG-PCL micelles in both creating an interaction with C6 rat glioma cells in vitro and delivering the therapeutic agent to the brain in a rat model of intractable malignant glioma, exhibiting high therapeutic efficacy after 7 days of continuous treatment [152] (Table 5). Kanazawa et al. [126] later tested the ability of the MPEG-PCL-TAT polymeric micelles to deliver to the brain siRNAs, characterized by a net negative charge and subject to excretion by the mucosa. They observed a significantly higher brain penetration following the intranasal administration of MPEG-PCL-TAT micelles when compared to IV administration in rats, thus confirming that the IN administration guarantees a more targeted delivery. Moreover, they observed that 15 min post-administration of fluorescein-labeled model siRNA (dextran, molecular weight: 10,000), the MPEG-PLG-TAT group exhibited a more intense fluorescence in the nasal mucosa than the naked group. Thus, they concluded that the MPEG-PLC-TAT micelles displayed a higher mucosal permeability. Investigations of the brain distribution after IN administration revealed that the MPEG-PLG-TAT group exhibited a significantly higher transfer to both the OB and the trigeminal nerve when compared to the naked group, suggesting that the use of CPP-modified polymeric micelles might enhance the delivery of nucleic acids to the brain via both the olfactory and trigeminal routes upon IN administration (Table 5). Furthermore, the distribution of the biotherapeutic to other brain areas such as the rostral brain tissue, caudal brain tissue, and brainstem was also demonstrated using MPEG-PLC-TAT/Alexa-dextran micelles upon IN administration. Kanazawa and colleagues [153] later investigated the therapeutic effects of IN administered MPEG-PCL-TAT co-loaded with the anti-cancer drug CPT and an anti-rat Raf-1 siRNA (siRaf-1) on a rat model of malignant glioma. Both MPEG-PLC-TAT and CPT-loaded MPEG-PLC-TAT were shown to form a stable complex with siRNAs and to be able to deliver siRaf-1 into cells in in vitro studies. Moreover, while both MPEG-PCL-TAT/siRaf-1 and CPT-loaded MPEG-PCL-TAT complexes alone determined cell death in vitro, a combination of the two was shown to significantly enhance the induction of cell death in rat glioma C6 cells. In addition, co-loading siRaf-1 and CPT into MPEG-PLC-TAT micelles, thus forming the CPT-loaded MPEG-PCL-TAT/siRaf-1 complex, produced even better results in vitro, inducing cell death to an even greater extent than the combination of MPEG-PCL-TAT/siRaf-1 and CPT-loaded MPEG-PCL-TAT complexes. Comparing the therapeutic efficacies of the IN administered siRaf-1 alone, MPEG-PCL-TAT/control siRNA, MPEG-PCL-TAT/siRaf-1 complex, CPT-loaded-MPEG-PCL-TAT micelles/control siRNA complex, and CPT-loaded MPEG-PCL-TAT micelles/siRaf-1 complexes in glioma model rats, the MPEG-PCL-TAT micelles were proven to be capable of enhancing both siRNAs and CPT delivery to the brain, thus positively contributing to increased survival when used with siRaf-1 and CPT individually or in combination (Table 5). The MPEG-PCL-TAT delivery agent was also safe, since no macroscopic signs of damage could be spotted in the rat nasal mucosa, brain tissue, and olfactory and trigeminal nerves.

The same N2B drug delivery system, composed of membrane-penetrating polymeric micelles, was also later tested for the delivery of an anti-tumor necrosis factor-α siRNA (siTNF-α) for the treatment of cerebral stroke. Its therapeutic effects were evaluated on a transient middle cerebral artery occlusion (t-MCAO) rat model of cerebral ischemia-reperfusion injury [154]. IN administration of siTNF-α/PEG-PCL-TAT complexes, 30 min after the induction of infarction, was shown to provide a shrinkage of nearly two-thirds of the infarcted area, to significantly suppress the production of the pro-inflammatory cytokine TNF-α, and to improve the neurology scores 22 h post-infarction when compared to the control groups (Table 5). These observations suggest that the N2B delivery of siTNF-α conjugated with PEG-PCL-TAT micelles is a valuable solution to alleviate the symptoms of cerebral ischemia-reperfusion injury.

More recently, another example of polymeric micelles functionalized with the addition of a CPP was provided by Yang et al. [130], who described a new type of nanomicelle for the IN delivery of siRNAs for the treatment of gliomas. This new delivery nanosystem is composed of a core, formed by the CPP DP7-C and the siRNA of interest, enveloped by a shell of hyaluronic acid (HA), to constitute a multifunctional “core-shell” nanomicelle. The advantage of these HA/DP7-C/siRNA nanomicelles is that they show strong stability and the capacity to protect the nucleic acid of interest. Moreover, the coating with HA has several advantages. Not only does it prolong the retention time of the drug, but it also prevents the drug from entering the lungs and improves mucosal permeability. Furthermore, HA binds to CD44 receptors, which are overexpressed on the membrane of many solid tumors, thereby improving accumulation of the therapeutic agent on glioma cell membranes. In in vitro studies conducted on GL261 cells, HA/DP7-C/siRNA nanomicelles showed high efficiency at transferring the siRNA into the cells, and low cytotoxicity and ability to escape from endosomes after internalization through macropinocytosis and clathrin-mediated endocytosis. Ex vivo bioimaging experiments conducted in mice showed the ability of HA/DP7-C/siRNA nanomicelles to deliver their content to the CNS and to accumulate in the brain within hours after a single IN administration. Interestingly, the therapeutic effects of HA/DP7-C micelles, loaded with vascular endothelial growth factor (VEGF)- or polo-like kinase 1 (PLK1)-specific siRNAs, were assessed in both in vitro experiments and in an intracranial glioma mouse model. HA/DP7-C/siVEGF or HA/DP7-C/siPLK1 showed promising results in vitro, determining (i) a decrease in the mRNA and protein levels of VEGF and PLK1, respectively, as well as in their downstream effectors, (ii) the suppression of angiogenesis for HA/DP7-C/siVEGF, and (iii) the induction of apoptosis for HA/DP7-C/siPLK1 nanosystems. The in vivo results were also promising, since the IN delivery of HA/DP7-C/siVEGF or HA/DP7-C/siPLK1 was reported to successfully downregulate the expression of the VEGF or PLK1 proteins while ensuring slower weight loss and prolongation of survival (Table 5). Moreover, the safety of the HA/DP7-C/siRNA nanomicelles was assessed in an in vivo toxicity study in rats. No significant toxic effect on vital organs, nasal mucocillary system, and trigeminal nerves were reported after a therapeutic time of administration.

Functionalization with CPPs has also been proposed for polymeric nanoparticles with the ultimate aim to improve their transmucosal transport, thus facilitating their N2B delivery. For instance, after functionalizing LMWP to the surface of poly(ethyleneglycol)-poly(lactic acid) (PEG-PLA) nanoparticles (LMWP-NPs), Xia et al. [155] extensively studied the brain delivery properties of the developed LMWP-NPs upon IN administration. They proved the usefulness of CPPs as delivery agents in the formulation of nanoparticles. LMWP-NPs were reported to show significantly enhanced cellular accumulation in vitro when compared to their unmodified counterpart as well as to enter the cell via both lipid raft-mediated endocytosis and direct translocation processes without any macroscopic cytotoxic effect. Moreover, in vivo studies conducted in rats revealed that IN administration of fluorescently-labelled coumarin-6-loaded LMWP-NPs enhanced the amount of coumarin delivered to different brain areas, with a significant increase in Cmax and AUC_0–8h_ 1 h after the administration in the cerebrum [Cmax 1053.1 pg/g vs. 493.7 pg/g; AUC_0–8h_ 5133.7 pg h/g vs. 2529.7 pg h/g], cerebellum [Cmax 1312.1 pg/g vs. 531.7 pg/g; AUC_0–8h_ 4352.3 pg h/g vs. 1706.9 pg h/g], olfactory tract [Cmax 825.5 pg/g vs. 287.9 pg/g; AUC_0–8h_ 2782.7 pg h/g vs. 1039.3 pg h/g], and OB [Cmax 1038.5 pg/g vs. 395.9 pg/g; AUC_0–8h_ 3538.8 pg h/g vs. 1251.4 pg h/g]. Additionally, although LMWP functionalization was shown to enhance the passage of coumarin in the systemic circulation, LMWP-NPs could obtain a much higher AUC_brain_/AUC_blood_ ratio than non-functionalized nanoparticles [cerebrum 3.230 vs. 2.725; cerebellum 2.738 vs. 1.839; olfactory tract 1.751 vs. 1.120; OB 2.226 vs. 1.348], suggesting that the LMWP modification can facilitate direct brain targeting.

Solid lipid nanoparticles have also been modified with CPPs in order to ameliorate their delivery to the brain via the IN route. An example of this approach comes from Rassu et al. [156], who proposed the use of the CPP RVG-9R in combination with chitosan-coated and -uncoated solid lipid nanoparticles for the delivery of a beta secretase BACE1 siRNA, potentially useful in the treatment of AD.

In vitro studies were performed to evaluate the permeation capacity of the siRNA alone and the siRNA released from chitosan-coated or -uncoated solid lipid nanoparticles using Caco-2 cells as a model for the nasal epithelium. Both formulations had the ability to increase the intracellular transport of the BACE1 siRNA, significantly enhancing the penetration of the siRNA through the model epithelial cells. Chitosan-coated nanoparticles were the most efficient, thereby proving that the developed nanosystem might be useful in the N2B delivery of siRNAs.

Finally, Samaridou and colleagues [157] recently proposed a novel delivery strategy that involved the combination of polymers, CPPs, and fatty acids, with the ultimate intent of overcoming the biological barriers and ensuring the efficient N2B delivery of the RNAs of interest including siRNAs. In particular, their approach involves the formation of electrostatically driven nanocomplexes between the CPP R8, modified with the fatty acid lauric acid (C12), and the RNA of interest. These nanocomplexes are then coated with protective polymers such as polyethylene glycol-polyglutamic acid (PEG-PGA) or HA. Hydrophobic modification of the CPPs, achieved by conjugation with fatty acids such as stearic, lauric, or myristic acids, results in increased lipophilicity that enhances both their stability and membrane penetration capacity [57,58]. These structures have already been tested in N2B delivery, showing some promising results [158]. On the other hand, the use of polymers is well-known to guarantee improved stability and protection against enzymatic degradation while facilitating the diffusion of the CPP-RNA nanocomplexes across the mucus layer. Related to this point, it is important to mention that while the C12-R8:RNA complexes were unable to protect the associated nucleic acid in phosphate buffered saline containing fetal bovine serum, the envelopment with both PEG-PGA or HA ensured protection and the absence of premature release in the presence of both enzymes and ions. C12-R8:RNA complexes were characterized by a positive charge and a diameter of about 69 nm, the PEG-PLA coated enveloped nanocomplexes (ENCPs) had a size of about 96 nm and a neutral charge, whereas the HA coated nanocomplexes (HA-ENCPs) had a size of 106 nm and a negative charge. In vitro studies performed with miR-132, a microRNA mimic with reported therapeutic action in AD, showed a significantly higher rate of cell internalization of the therapeutic agent when administered associated with the ENCPs, particularly PEG-PGA ENCPs [~11,000-fold change for HA-ENCPs; ~14,000-fold change for PEG-PGA ENCPs], when compared to free miR-132 complexed with the RNAiMAX lipofection reagent. This evidence confirms the ability of these delivery agents to induce cellular uptake. The brain distribution and therapeutic effects in vivo of the PEG-PGA ENCPs were also evaluated in a mouse model of AD upon IN administration. IN administered miR-132-loaded PEG-PGA ENCPs were found to be capable of delivering the RNA in its active form to the hippocampus through the N2B route, resulting in an increased level of miR-132 and in the downregulation of two predicted target mRNAs [157] (Table 5).

## 14. Concluding Remarks

On the bases of the observations described in the previous paragraphs, we can conclude that CPPs have proven to be valuable tools for the delivery of biotherapeutics in different circumstances. More specifically, CPPs have the ability to permeate cell membranes, biological barriers (e.g., skin, conjunctiva of the eye, and BBB), and tissues [4]. Moreover, they can be conjugated to a variety of biologically active molecules and thus be used as vehicles to deliver their cargoes into cells. CPPs can enter the cells in a non-invasive manner (i.e., preserving the membrane integrity) [26], effectively enhancing the cell permeability of their cargo molecules [4]. In an attempt to introduce biotherapeutics to the CNS, CPPs have been proposed as delivery agents to permeate the BBB, which represents the major obstacle to CNS delivery. In spite of the promising results in terms of CNS delivery obtained upon the systemic administration of CPP–cargo complexes in both preclinical and clinical studies [66,67,68,69,70], one issue that remains to be solved is the low tissue selectivity of most peptides, which may cause the accumulation of the CPP–drug complex in peripheral organs, thereby causing secondary effects [22]. Thus, alternative administration routes have been explored in order to defeat this problem including the IN route, which represents a non-invasive way to achieve direct drug delivery to the CNS while reducing systemic exposure [9]. However, despite the numerous advantages of N2B delivery, limitations have also emerged. The principal drawback is that only a small volume of therapeutic can be administered at once, making this administration route only suitable for highly potent drugs [71,73]. Furthermore, to achieve efficient delivery to the CNS via IN administration, it is extremely important that the therapeutic has the capacity to permeate the nasal epithelia [80,81]. Unfortunately, in most cases, biotherapeutics struggle to penetrate this tissue. Thus, they may need up to 10-fold higher doses when compared to the IV route of administration to achieve comparable drug levels in the CNS [20]. Thus, the use of penetration enhancers, particularly CPPs, have been proposed to increase the passage through the nasal epithelium. CPPs, both individually and in combination with polymers and/or lipids to create nanometric drug carriers, are currently being tested as IN delivery agents in in vitro and in vivo experiments. CPPs and CPP-functionalized nanocarriers have been proven to be capable of enhancing the IN delivery of biotherapeutics, delivering the intact therapeutic agent to the CNS where it can exert its therapeutic function as such or in association with rehabilitative training to maximize recovery, particularly in cases where brain areas controlling motor skills, speech, vision, and thinking are affected by the injury/disease.

## Figures and Tables

**Table 1:** Classification of CPPs.

Type of Classification	Categories	Examples	Refs.
Based on physical–chemical properties	Cationic peptides	TAT-derived moieties, penetratin, polyarginines, LMWP, crotamine	[4,24,25]
	Amphipathic peptides	MPG, Pep-1, MAP, transportan, azurin-derived p28 peptide, proline-rich CPPs	[4]
	Hydrophobic peptides	C105Y and derivatives, Pep-7	[4]
Based on type of coupling to the cargo	Covalently bound	TAT derivatives, penetratin, and polyarginines	[24,26]
	Non-covalently bound	Pep, MPG, KALA, KLA and PepFect types	[24,26]
Based on uptake mechanism	Membrane translocation	Transportan analogs, MPG, Pep-1, dermaseptin	[4,22,26,31]
	Endocytosis	TAT derivatives, polyarginines, transportan-based complexes, NickFect1, proline-rich CPPs, azurin-derived peptides, LMWP	[32,33,34,35,36,37,38]

**Table 2:** List of nasal drugs put on the market for the treatment of CNS disorders.

Drug	Brand Name	Indications	Manufacturer	Status	Refs.
Dihydroergotamine mesylate (DHE-45)	Migranal^®^	Migraine	BAUSCH	Available	[83]
Desmopressin acetate	Stimate^®^	Hemophilia A	Ferring Pharmaceuticals US	Voluntary recall *	[84]
Nafarelin acetate	Synarel^®^	Central precocious puberty	Pfizer	Available	[85]
Butorphanol tartrate	Stadol^®^	Migraine and pain	Bristol Myers Squibb	Discontinued *	[86]
Zolmitriptan	Zomig^®^	Migraine	Astra Zeneca	Available	[87]
Desmopressin acetate	DDAVP^®^	Prevention of polydipsia and polyurea, head trauma	Ferring Pharmaceuticals US	Voluntary recall *	[88]
Esketamine	Spravato^®^	Resistant depression	Janssen-Cilag S.p.a.	Available	[89]
Midazolam	Nayzilam^®^	Seizure clusters	UCB pharmaceutics	Available (only in USA)	[90,91]
Diazepam	Valtoco^®^	Seizure clusters	Neurelis INC	Available (only in USA)	[92]

* Stimate^®^, Stadol^®^, and DDAVP^®^ results discontinued from the market. In August 2020, Ferring Pharmaceuticals US voluntarily recalled all lots on the market of DDAVP ^®^ Nasal Spray and STIMATE^®^ Nasal Spray due to superpotency or out-of-specification amounts of desmopressin reported during routine testing. Stadol^®^ was proven to determine a narcotic effect, leading to cases of addiction that caused death.

**Table 3:** Strategies proposed to increase the brain bioavailability of IN products.

Strategies to Increase Brain Bioavailability		Examples	Refs.
Enhancing drug solubility in the nasal cavity	Encapsulation complexes	IN delivery to the brain of GALP improved to threefold by encapsulation in cyclodextrins.	[113]
	Microemulsion and nanoemulsion formulations	IN emulsion-like formulation improved delivery of GDF5 to all regions of the CNS and to trigeminal nerve.	[80,115]
Reducing clearance, prolonging the residence time of the formulation at the delivery site.	Mucoadhesive and viscosity enhancing agents	Retention time and cell permeability of buspirone significantly increased in the rat nasal compartment, when formulated with 1% chitosan.	[116]
	Mucoadhesive and viscosity enhancing agents and encapsulating agents	IN delivery of buspirone resulted in twofold higher brain AUC when formulated with 1% chitosan and 5% hydroxypropyl β-cyclodextrin compared to the simple solution.	[117]
	Reversible and irreversible ciliostatics and ciliotoxic drugs	Ciliostatics impair ciliary movement and decrease mucus clearance. Ciliotoxic drugs cause damage to the cilia or epithelium by destroying their structure or integrity.	[81]
	Biogels	IN administration of rufinamide in xyloglucan-based, heat triggered biogels resulted in higher brain AUC compared to the simple suspension.	[118]
Reducing clearance due to efflux transporters or absorption by the nasal vasculature.	Transporter inhibitors	The use of transporter inhibitors such as rifampin resulted in greater brain uptake of several drugs.	[119,120]
	Vasoconstrictors	The addition of phenylephrine to IN formulations of hypocretin-1 or L-Tyr-D Arg resulted in a reduction in the amount of drug adsorbed into the blood and an increase in the amount delivered to the OB.	[121]
Reducing degradation by enzymes and proteases in the nasal cavity	Enzyme inhibitors	The use of P-glycoprotein inhibitors, CYP450 inhibitors. or acetazolamide reduced degradation and increased the amount of drug transported from the nasal compartment to the brain.	[81]

**Table 4:** The main results obtained in preclinical studies after IN delivery of CPP-conjugated biotherapeutics to the CNS.

Biotherapeutic/Pathology	CPP Conjugated	Results	Refs.
Insulin/Alzheimer’s disease	Non-covalently conjugated L-/D-penetratin	-Both peptides enhanced insulin levels in OB. -L-penetratin increased insulin concentrations in the hypothalamus, cerebral cortex, cerebellum, and brain stem. -L-penetratin increased insulin levels in plasma (systemic side effects). -D-penetratin maintained a more favorable AUC_brain_/AUC_blood_ ratio	[20,132]
Insulin/Dementia	Non-covalently conjugated L-/D-penetratin	-L-penetratin enhanced IN delivery of insulin. -In early stage dementia, L-penetratin contributed to ameliorate the therapeutic action of insulin, although causing an undesirable hypoglycemic effect.	[131]
Exendin-4/Dementia	Non-covalently conjugated L-penetratin	-L-penetratin facilitated N2B delivery of exendin-4, increasing its concentration in hypothalamus, hippocampus, cerebral cortex, cerebellum, and brain stem. -Co-administration of exendin-4 and L-penetratin only slightly improved progressive cognitive dysfunction in a senescence-accelerated mouse model (SAMP8 mice).	[133]
Exendin-4 + Insulin/Dementia	Non-covalently conjugated L-penetratin	-Co-administration of exendin-4 and low-dose insulin with L-penetratin improved cognitive and spatial functions in SAMP8 mice.	[133]
haFGF/Alzheimer’s disease	Covalently-bound TAT	-TAT conjugation enhanced delivery and distribution of haFGF to the brain. -Favorable brain to blood ratio is maintained. -IN TAT-haFGF was more effective than the IV administration in increasing ACh levels, improving learning and memory abilities, and reducing the number and size of Aβ plaques in the AβPP/PS1 AD mouse brain.	[138,139]
haFGF/Dementia	Covalently-bound TAT	-TAT-haFGF treatment corrected cholinergic deficits, decreased the amount of Aβ deposits, and decreased the number of apoptotic neurons and oxidative stress in SAMP8 mice. -Improved learning and memory abilities of SAMP8 mice.	[138]
Leptin/Obesity	Non-covalently conjugated L-penetratin	-The treatment causes stimulation of leptin receptors and activation of Stat3 effector, with consequent reduction in the plasma triglycerides and decreased body weight.	[136]
NMU/Inflammation-mediated amnesia	PAS-R8 F4-R8	-PAS-R8-NMU is more stable and more efficiently delivered to the CNS than F4-R8-NMU. -While both IN-administered NMU derivatives prevented or reduced LPS-induced memory impairment, IN NMU alone did not.	[137]
GLP-2/Major depression	PAS-R8	-Both the CPP and the PAS portions are essential to ensure GLP-2 access to the brain upon IN administration and to induce an antidepressant-like effect. -The systemic absorption of PAS-CPP-GLP-2 was extremely low.	[134,135]
BSA	Covalently-bound LMWP	-Conjugation with LMWP gives BSA the ability to reach the CNS (OB) and achieve deep inward penetration.	[125]
HRP β-gal	Covalently-bound LMWP	-Conjugation with LMWP enhanced the retention of enzymatic activity of the two proteins in the CNS.	[125]

**Table 5:** The CPP-coupled nanocarriers proposed for the N2B delivery of therapeutics.

Nanocarrier	Pharmacological Agent	Results	Ref.
MPEG-PLC-TAT polymeric micelles	Coumarin	-Brain distribution of coumarin, 4 h after IN administration, was higher with MPEG-PLC-TAT micelles than with the MPEG-PLC micelles. -Concentration of coumarin in non-targeted tissues was lower when administered with MPEG-PLC-TAT compared to the solution alone.	[55]
	Camptothecin	-MPEG-PLC-TAT micelles were more effective than the MPEG-PLC micelles in ensuring an interaction with glioma cells and delivering the agent to the rat brain.	[152]
	siRNAs	-MPEG-PLC-TAT guaranteed a better mucosa permeability and a higher transfer to the OB and trigeminal nerve when compared to the naked siRNA.	[126]
	Camptothecin+siRaf-1	-MPEG-PLC-TAT micelles enhanced siRaf-1 and camptothecin delivery to the brain both individually or in combination	[153]
	siTNF-α	-This system provided shrinkage of the infarcted area, suppression of TNF-α production, and improvement in the neurology scores in the t-MCAO rats	[154]
HA/DP7-C polymeric micelles	siVEGF siPLK1	-In glioma cells, these systems provided the downregulation of VEGF and PLK1 mRNA and protein levels, suppression of angiogenesis for the siVEGF system and induction of apoptosis for the siPLK1 system. -In mice, the IN administration of these systems downregulated the VEGF and PLK1 protein levels and prolonged survival.	[130]
PEG-PLA nanoparticles functionalized with LMWP	Coumarin	-These systems enhanced cellular accumulation in vitro when compared to non-CPP-coupled nanoparticles. -These nanoparticles increased the amount of drug delivered to different brain areas in vivo, exhibited higher Cmax and AUC_0–8h_, and showed enhanced AUC_brain_/AUC_blood_ ratio than non-functionalized nanoparticles.	[155]
RVG-9R- chitosan coated/uncoated solid lipid nanoparticles	BACE1 siRNA	-Both formulations enhanced the penetration of the siRNA through an in vitro model of the nasal epithelium.	[156]
C12-R8 PEG-PGA nanocomplexes	miR-132	-These nanocomplexes increased the rate of internalization of the therapeutic agent in vitro. -These systems were capable of delivering miR-132 to the hippocampus, increasing the level of miRNA-132, and downregulating the target mRNAs.	[157]

## Data Availability

Not applicable.
